# TOPK promotes immune suppression in kidney renal clear cell carcinoma and emerges as a prognostic and therapeutic target

**DOI:** 10.1186/s12885-025-14665-0

**Published:** 2025-08-18

**Authors:** Zeyuan Zheng, Renhui Xiong, Xiuyuan Sui, Liyan Li, Huimin Sun, Chen Shao

**Affiliations:** 1 Department of Urology, Xiang’an Hospital of Xiamen University, School of Medicine, Xiamen University, Xiamen, 361101 China; 2 Department of Oncology, Xiang’an Hospital of Xiamen University, School of Medicine, Xiamen University, Xiamen, 361101 China; 3 Central Laboratory, Xiang’an Hospital of Xiamen University, School of Medicine, Xiamen University, Xiamen, 361101 China

**Keywords:** T-LAK cell-originated protein kinase (TOPK), Tumor microenvironment (TME), Biomarkers, Immunotherapy, Kidney Renal Clear Cell Carcinoma (KIRC)

## Abstract

The T-LAK cell-originated protein kinase (TOPK) has been associated with poor prognosis in various cancers, yet its specific role in kidney renal clear cell carcinoma (KIRC) remains unclear. In this study, we conducted a comprehensive investigation of TOPK expression and its association with immune infiltration in KIRC by integrating bioinformatics, in vitro, and in vivo approaches. We found that TOPK is significantly overexpressed in KIRC and serves as an independent prognostic marker correlated with aberrant promoter methylation. High TOPK expression was associated with reduced infiltration of cytotoxic immune cells and increased immune checkpoint expression, indicating its potential role in shaping an immunosuppressive tumor microenvironment (TME). Functional enrichment analysis further linked TOPK to key inflammatory signaling pathways, including NOTCH1, TNF-α, and TGF-β. In vivo experiments using TOPK-deficient tumor models in immunocompetent mice supported its role in modulating tumor-associated inflammation.While these experiments were based on a non-renal tumor model, the findings nonetheless provide meaningful insights into the immunoregulatory function of TOPK in vivo. Collectively, our results identify TOPK as a promising prognostic biomarker and immunotherapeutic target in KIRC, and offer novel perspectives on its involvement in immune evasion mechanisms.

**Supplementary Information:**

The online version contains supplementary material available at 10.1186/s12885-025-14665-0.

## Background

Kidney Renal Clear Cell Carcinoma (KIRC) represents approximately 3% of all cancer cases, with the highest incidence observed in western nations. The only curative treatment for localized KIRC remains surgical intervention [1]. Presently, clinical treatments primarily include targeted therapies, such as vascular endothelial growth factor inhibitors/tyrosine kinase inhibitors and immunotherapy. However, among the initial responders to such treatments, approximately 70% of them eventually develop resistance, with 30% displaying inherent resistance to VEGF-TKIs [2], and has been intricately linked with high tumor heterogeneity and the tumor microenvironment (TME) [3].

An increasing number of studies have highlighted the pivotal role that the TME plays in governing tumor growth and metastasis and influencing the efficacy of immunotherapy [4]. Tumor-infiltrating immune cells are integral components of the TME, and while there has been some elucidation of the overall tumor immune microenvironment in renal cancer [5], there is still a need for further investigation into specific biomarkers. In addition, strategies aimed at reprogramming the TME and reversing immune suppression may yield valuable insights for enhancing current cancer treatment modalities.

The emergence and progress of immunotherapy have significantly advanced cancer treatment strategies, particularly in the context of tumor immunology [6]. Current research is diligently advancing investigations regarding the combination of anti-angiogenic therapies with immunotherapeutic drugs in KIRC, with the aim to elucidate the complexities of the renal cancer immune microenvironment and gain more insights into immune infiltration, which could be essential for improving treatment response rates and developing new personalized immunotherapy strategies to enhance patient prognoses.

T-LAK cell-originated protein kinase (TOPK) is a pivotal protein kinase with a multifaceted role in various biological processes, including but not limited to cell proliferation, cell cycle regulation, apoptosis, DNA damage repair, and cellular drug resistance [7–9]. Recent research has provided compelling evidence of high TOPK expression in various human malignancies, with clear associations to adverse prognoses in multiple cancer types [10–12]. Previous investigations revealed that TOPK undergoes phosphorylation and activation at the Ser 32 site by ERK2, which contributes to resistance against sunitinib in KIRC. Furthermore, the combination of a TOPK inhibitor with sunitinib has been shown to enhance the sensitivity of sunitinib-resistant renal cancer cells, suggesting that phosphorylated TOPK could be a promising biomarker for predicting sunitinib resistance [13].

Nonetheless, the current focus on TOPK in cancer predominantly revolves around its functions within tumor cells, with limited attention to its role within the TME. Our recent research offers additional evidence that TOPK can upregulate PD-L1 expression in KIRC, consequently influencing the cytotoxicity of CD8 + T cells and facilitating immune evasion [14]. These findings indicate the important association between TOPK and the immune microenvironment within renal cancer tumors.

The role of TOPK in modulating the tumor immune microenvironment (TME) remains poorly understood. Here, large-scale bioinformatics analyses identified high TOPK expression as an independent prognostic factor in renal cancer, associated with increased infiltration of anti-tumor immune cells yet contributing to an immunosuppressive TME. In TOPK knockout tumor-bearing mice, inflammation-related pathways were upregulated, suggesting that TOPK promotes immunosuppressive TME formation by regulating inflammatory signaling. These findings lay the groundwork for developing TOPK-targeted cancer immunotherapies.

## Results

### Result 1: Comprehensive analysis of TOPK expression patterns in KIRC

**Fig. 1:**
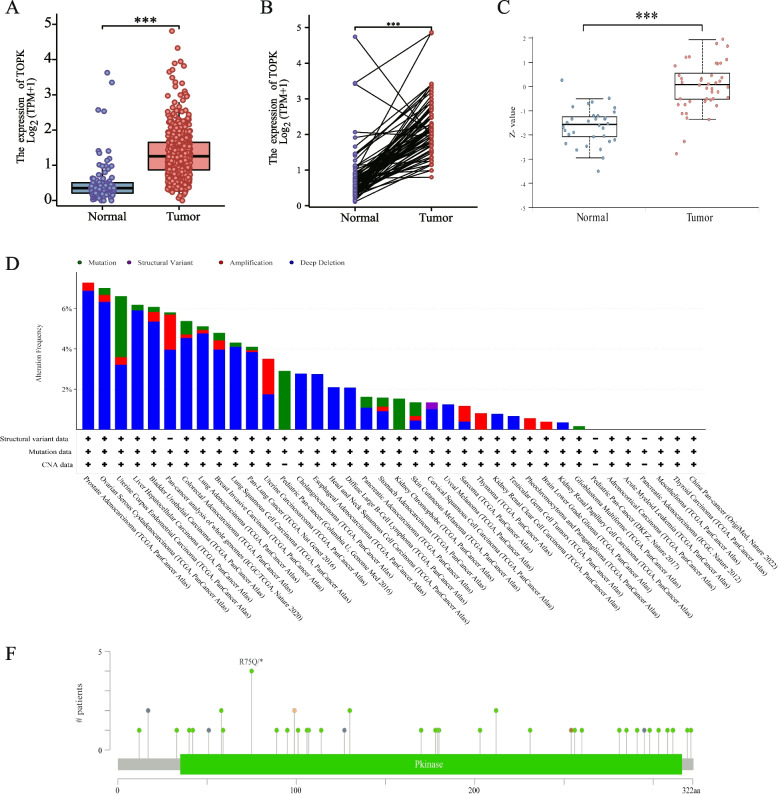
TOPK expression in KIRC. **A** Expression of TOPK in tumor and normal tissues in TCGA + GETx dataset. **B** Expression of TOPK in tumor and paired normal tissues samples. **C** Protein levels of TOPK in KIRC and normal tissues samples. **D** Scatter plot of TOPK mRNA and copy-number alterations in KIRC. **E** TOPK mutation frequency in multiple TCGA pan-cancer dataset in the cBioPortal database. **F** Mutation sites of TOPK across protein domains in different cancer types. *p < 0.05; **p < 0.01; ***p < 0.001

Transcriptomic profiling of TOPK mRNA expression levels in KIRC patients derived from The Cancer Genome Atlas (TCGA) database revealed significant dysregulation of TOPK across multiple malignancies when compared with matched normal tissue controls (Fig. S1A). Notably, TOPK exhibited pronounced transcriptional upregulation in several cancer types, suggesting its potential oncogenic role in tumorigenesis… Systematic paired-sample analysis demonstrated statistically significant overexpression of TOPK mRNA in tumor tissues across 11 cancer types, including bladder urothelial carcinoma (BLCA), colon adenocarcinoma (COAD), head and neck squamous cell carcinoma (HNSC), hepatocellular carcinoma (LIHC), lung adenocarcinoma (LUAD), lung squamous cell carcinoma (LUSC), prostate adenocarcinoma (PRAD), stomach adenocarcinoma (STAD), thyroid carcinoma (THCA), and uterine corpus endometrial carcinoma (UCEC), (Fig. S1B), with particularly robust upregulation observed in KIRC patients (Fig. 1A, B).

Immunohistochemical analysis of TOPK protein expression patterns revealed strong concordance with transcriptional data. Notably, KIRC patients exhibited markedly elevated TOPK protein expression compared to normal renal parenchyma, further corroborating the transcriptome-level findings. This consistent overexpression at both mRNA and protein levels suggests potential functional significance of TOPK in renal carcinogenesis and warrants further mechanistic investigation (Fig. 1C).

### Result 2: Methylation level and mutation analysis of TOPK in KIRC

To gain further insights into the mechanisms governing TOPK gene expression in KIRC, we first investigated the mutation status of TOPK in KIRC because of the intricate relationship between gene mutations and gene expression [15, 16]. Next, we performed a comprehensive analysis of TOPK mutations in 38 KIRC datasets (TCGA, Firehose Legacy) using the cBioPortal database. Our results revealed that TOPK alterations across multiple cancer types primarily consisted of mutations, structural variants, amplifications, and deep deletions. Additionally, the key copy number variations (CNVs) affecting TOPK included deep deletion, shallow deletion, diploid, and gain, with diploid status showing the strongest association with TOPK mutation frequency (Fig. 1D, E). In the wider context of cancer, the primary mutation sites observed in TOPK were predominantly located at R75Q/* (Fig. 1F). R75 (arginine 75) is located in the N-terminal functional domain of TOPK and may be involved in protein–protein interactions or kinase activity regulation. Arginine (R) carries a positive charge. Its mutation to glutamine (Q) may cause local charge or conformational changes, affecting the binding of TOPK to substrates or other proteins.However, it is crucial to emphasize that TOPK mutations were relatively rare in KIRC.

**Fig. 2:**
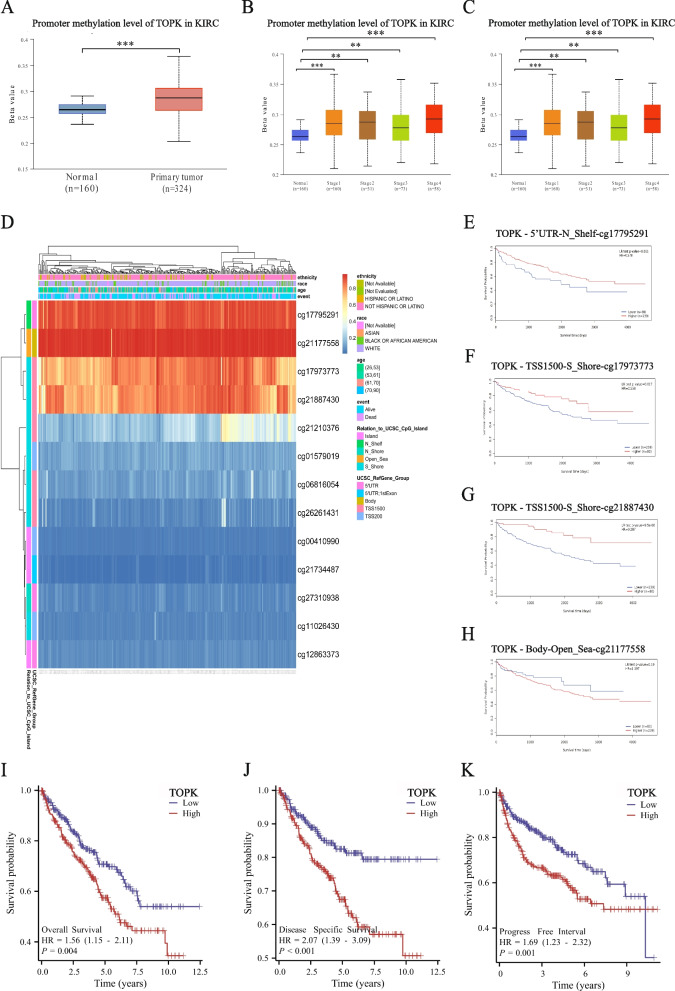
DNA methylation in TOPK. **A** Promoter methylation level of TOPK in KIRC. **B** Correlation of promoter methylation level of TOPK and stage. **C** Correlation of promoter methylation level of TOPK and grade. **D** Heatmap of cluster analysis for a single CpG in TOPK. **E**–**H** Prognosis of different CpG sites in patients with KIRC. **I**-**K** Relationship between TOPK expression and patient prognosis (OS, DSS and PFI) of KIRC in TCGA database.*p < 0.05; **p < 0.01; ***p < 0.001

DNA methylation regulation is commonly associated with gene expression. It is widely accepted that high methylation at gene promoters is correlated with the suppression of gene transcription, whereas methylation of CpG islands can promote gene transcription [17, 18]. Here, we analyzed TOPK's promoter methylation levels in the UALCAN database, which revealed that in KIRC patients, the TOPK promoter methylation levels were significantly higher than those observed in the normal group (Fig. 2A). Further investigations on the levels of TOPK promoter methylation in different tumor stages and grading subgroups of KIRC patients demonstrated that the methylation levels of the TOPK promoter also increased with advanced tumor stage and grade (Fig. 2B, C).

Subsequently, we used the MethSurv tool to assess the CpG island methylation status of the TOPK gene and identified four sites, namely cg17795291, cg21177558, cg17973773 and cg21887430, as the top four sites with the highest levels of CpG island methylation (Fig. 2D). Further investigation revealed that KIRC patients with high CpG island methylation at cg17795291, cg17973773 and cg21887430 exhibited more favorable prognoses, while those with lower methylation levels had poorer outcomes. Notably, cg21177558 did not demonstrate a statistically significant impact on prognosis (Fig. 2E-H).

### Result 3: TOPK is associated with poor prognosis in KIRC

Survival analysis further elucidated the prognostic significance of TOPK expression in KIRC patients. Notably, elevated TOPK expression was associated with worse clinical outcomes, demonstrating significantly shorter overall survival (OS)(HR: 1.56, 95% CI: 1.15–2.11; *P* = 0.004, Fig. 2I), disease-specific survival (DSS) (HR: 2.07, 95% CI: 1.39–3.09; *P* < 0.001, Fig. 2J), and progression-free survival (PFS) (HR: 1.69, 95% CI: 1.23–2.32; *P* = 0.001, Fig. 2K), compared to patients with lower TOPK levels.

Furthermore, TOPK expression levels were significantly correlated with key clinicopathological characteristics in KIRC patients (Table 1). Specifically, TOPK overexpression showed strong associations with advanced tumor stage (T stage), nodal involvement (N stage), distant metastasis (M stage), higher pathologic stage, aggressive histologic grade, and adverse clinical outcomes (OS, DSS, and PFI events).

**Table 1:** Correlation between TOPK and clinicopathological characteristics in patients with KIRC

Characteristics	Low expression of TOPK	High expression of TOPK	P value
**n**	270	271	
Age, n (%)			0.699
< = 60	132 (24.4%)	137 (25.3%)	
> 60	138 (25.5%)	134 (24.8%)	
Pathologic T stage, n (%)			< 0.001
T1&T2	195 (36%)	155 (28.7%)	
T3&T4	75 (13.9%)	116 (21.4%)	
Pathologic N stage, n (%)			0.006
N0	116 (45%)	126 (48.8%)	
N1	2 (0.8%)	14 (5.4%)	
Pathologic M stage, n (%)			0.002
M0	224 (44.1%)	205 (40.4%)	
M1	26 (5.1%)	53 (10.4%)	
Pathologic stage, n (%)			< 0.001
Stage I&Stage II	186 (34.6%)	146 (27.1%)	
Stage III&Stage IV	83 (15.4%)	123 (22.9%)	
Histologic grade, n (%)			0.005
G1&G2	141 (26.5%)	109 (20.5%)	
G3&G4	125 (23.5%)	158 (29.6%)	
OS event, n (%)			0.001
Alive	200 (37%)	166 (30.7%)	
Dead	70 (12.9%)	105 (19.4%)	
DSS event, n (%)			< 0.001
No	228 (43%)	193 (36.4%)	
Yes	36 (6.8%)	73 (13.8%)	
PFI event, n (%)			0.002
No	206 (38.1%)	173 (32%)	
Yes	64 (11.8%)	98 (18.1%)	

### Result 4: Functional enrichments of TOPK-associated genes in KIRC

**Fig. 3:**
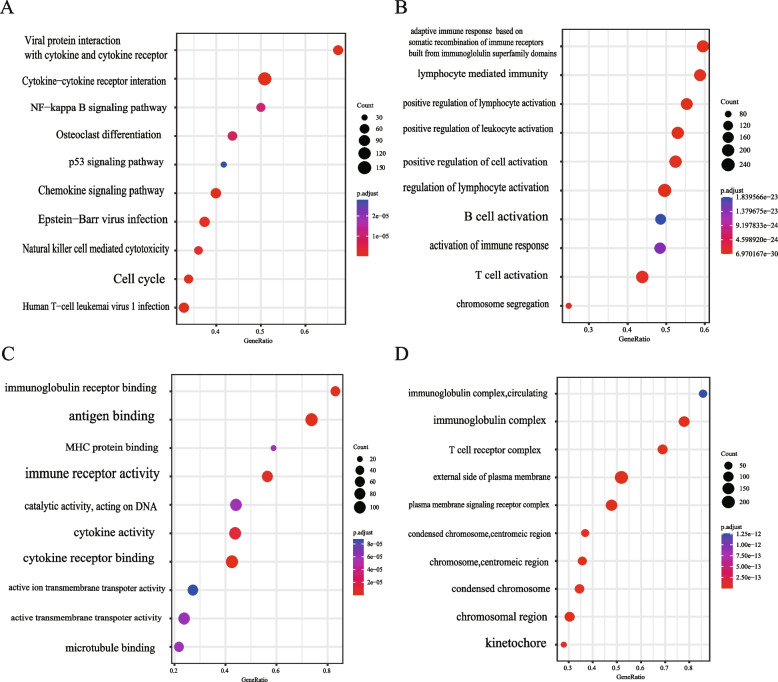
Enrichment analysis with TOPK. **A**-**C** GO analysis with TOPK (ALL, BP, MF, CC). **D** KEGG analysis with TOPK

The above findings suggest that TOPK could be a promising therapeutic target in renal cancer. To investigate the role of TOPK in tumor progression, we categorized patients based on their median TOPK expression levels and conducted differential analysis on the resulting set of differentially expressed genes, as well as Gene Ontology (GO) and Kyoto Encyclopedia of Genes and Genomes (KEGG) enrichment analyses. The KEGG results revealed significant associations between TOPK and several key signaling pathways, including those involved in neutrophil extracellular trap formation, Th17 cell differentiation, the T cell receptor signaling pathway, Natural killer cell-mediated cytotoxicity, NF-kappa B signaling pathway, p53 signaling pathway, Chemokine signaling pathway, and Cell cycle (Fig. 3A-C). The GO results highlighted TOPK's involvement in various aspects of the immune system, encompassing functions related to the immunoglobulin complex, T cell receptor complex, adaptive immune response driven by somatic recombination of immune receptors built from immunoglobulin superfamily domains, lymphocyte-mediated immunity, positive regulation of lymphocyte activation, positive regulation of leukocyte activation, and T cell activation (Fig. 3D). Collectively, these findings highlight the significant interplay between TOPK and the immune microenvironment in KIRC.

### Result 5: TOPK is associated with the immune microenvironment of KIRC

**Fig. 4:**
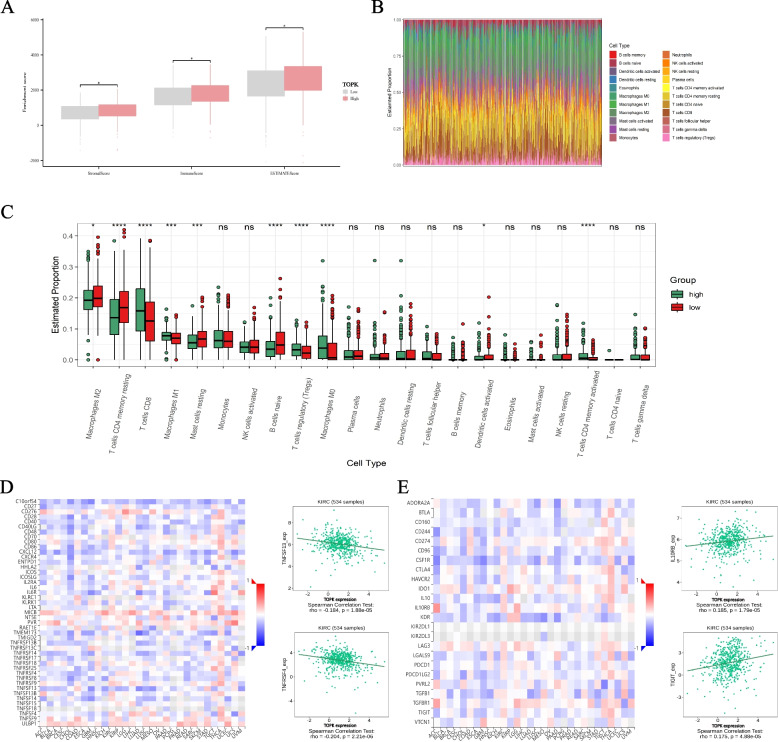
Immune infiltration in TOPK. **A** The ESTIMATE algorithm was used to evalue immune infiltration in patients with IRC. **B**-**C** The CIBERSORT algorithm was used to evalue immune infiltration in patients with KIRC. **D**-**E** Asscioation of immune-modulator and TOPK. *, *P* < 0.05. **, *P* < 0.01. ***, *P* < 0.001. ****, *P* < 0.0001

In recent years, significant progress has been made in the field of immunotherapy for renal cancer, maintaining its status as a central focus of research [19]. To further investigate whether TOPK expression is associated with the immune microenvironment of KIRC, we used the ESTIMATE algorithm to examine the correlation between TOPK and immune scores, stromal scores, and estimate scores within KIRC. The results indicate a positive correlation between TOPK and immune scores (Fig. 4A).

Subsequently, we further analyzed the relationship between TOPK and immune cells (Fig. 4B, C), which indicated that TOPK could influence the extent of immune cell infiltration in the immune microenvironment of KIRC, affecting various immune cell types, such as T cells, NK cells, and macrophages. Moreover, we identified a negative correlation between TOPK and immune stimulators (Fig. 4D), such as TNFSF13B (rho = −0.217, *P* < 0.001) and TNFRSF4 (rho = −0.204, *P* < 0.001). Additionally, the expression level of TOPK was found to be positive with immune suppressors (Fig. 4E), including IL10RB (rho = 0.185, *P* < 0.001) and IL10 (rho = 0.173, *P* < 0.001).

### Result 6: Correlation between TOPK expression and immune markers in renal cell carcinoma

**Fig. 5:**
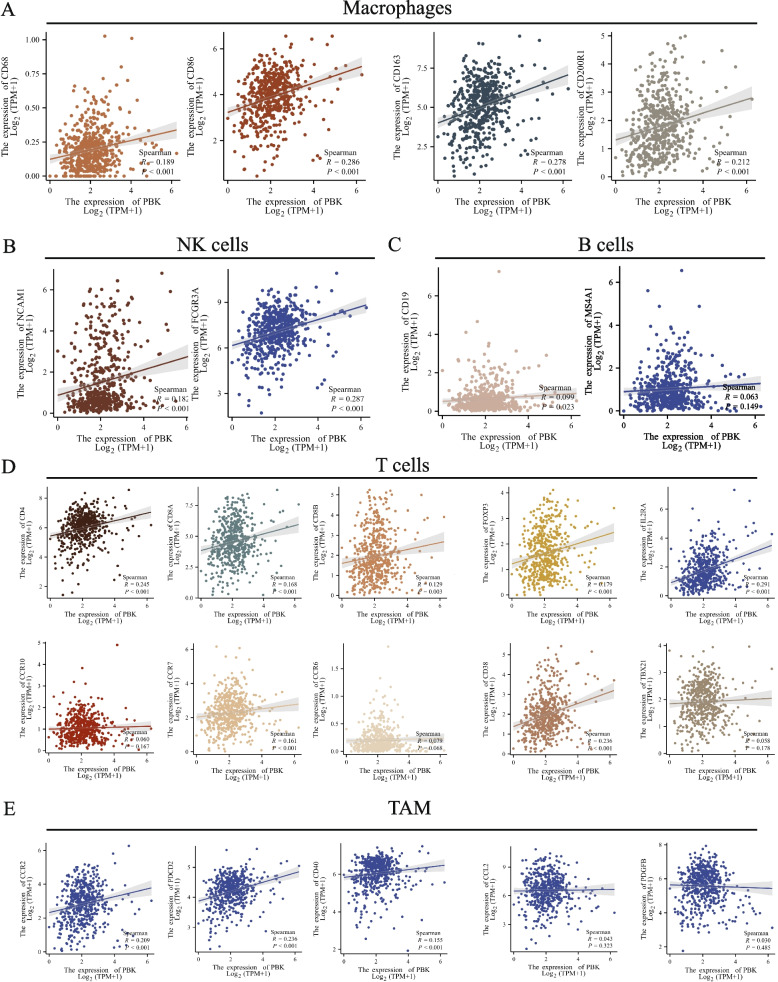
TOPK expression positively correlates with macrophages, NK cells, T cells, and TAMs, suggesting its potential role in promoting immunosuppressive cell infiltration in KIRC. **A** Correlation between TOPK and macrophage markers (CD68, CD86, CD163, CD200R1). **B** Correlation between TOPK and NK cell markers (NCAM1, FCGR3A, MS4A1). **C** Correlation between TOPK and B cell markers (e.g., CD38, CCR10, TBX21). **D** Correlation between TOPK and T cell markers (CD4, CD8A, CD8B, FOXP3, IL2RA, etc.). **E** Correlation between TOPK and tumor-associated macrophage (TAM) markers (CCR2, PDCD2, CD40, CCL2, PDGFB)

To further elucidate the relationship between TOPK and tumor-infiltrating immune cells, we performed correlation analyses between TOPK expression and established immune cell markers in KIRC. This comprehensive evaluation included markers for various immune cell subtypes, enabling systematic characterization of the immune microenvironment associated with TOPK expression. (Table 2) and found that the expression of TOPK was significantly correlated with markers of infiltrating lymphocytes, including CD8 + T cells, regulatory T cells (Tregs), exhausted T cells, as well as with markers of macrophages, tumor-associated macrophages (TAMs), and myeloid-derived suppressor cells (MDSCs). These findings further strongly suggest that the signaling pathways associated with TOPK may play a role in mediating the infiltration of immunosuppressive cells in KIRC (Fig. 5).

**Table 2:** Correlations between TOPK and gene markers of infiltrating immune cells

Cell Type	Gene Markers	None Cor	p		Purity Cor	p
**CD8 + Tcell**	CD8A	0.147	***		0.151	**
	CD8B	0.102	*		0.103	*
**T cell (general)**	CD3D	0.120	**		0.114	*
	CD3E	0.105	*		0.096	*
	CD2	0.165	***		0.163	***
**B cell**	CD19	0.081	0.063		0.066	0.159
	CD79A	0.008	0.855		−0.004	0.931
	CD27	0.091	*		0.086	0.065
**Monocyte**	CD14	0.124	**		0.112	*
	CSF1R	0.161	***		0.154	***
**TAM**	CD68	0.249	****		0.26	****
	CD11b	0.189	****		0.172	***
**M1 macrophage**	NOS2	0.089	*		0.066	0.158
	IRF5	0.15	***		0.164	***
	PTGS2	0.154	***		0.156	***
**M2 macrophage**	MRC1	0.183	****		0.191	****
	Dectin-1	0.194	****		0.196	****
	CD163	0.235	****		0.233	****
**Neutrophil**	CEACAM8	0.026	0.542		0.059	0.209
	ITGAM	0.189	****		0.172	***
	CCR7	0.125	**		0.127	**
**Natural killer cell**	KIR2DL1	−0.018	0.674		−0.024	0.609
	KIR2DL3	−0.004	0.932		0	0.996
	KIR2DL4	0.02	0.638		0.01	0.822
	KIR3DL1	−0.037	0.395		−0.019	0.688
	KIR3DL2	−0.074	0.0872		−0.069	0.138
	KIR3DL3	−0.008	0.853		−0.002	0.971
	KIR2DS4	−0.078	0.0714		−0.074	0.114
**Dentritic cells**	CD1C	0.089	*		0.084	0.072
	NRP1	0.137	**		0.128	**
	ITGAX	0.106	*		0.109	*
**Th1**	TBX21	0.038	0.384		0.033	0.479
	STAT4	0.166	***		0.169	***
**Th2**	GATA3	−0.064	0.142		−0.073	0.119
	STAT6	0.023	0.604		0.036	0.435
**Tfh**	BCL6	0.035	0.414		0.042	0.368
	IL21	0.153	***		0.142	**
**Th17**	IL17A	−0.037	0.392		−0.053	0.255
**Treg**	FOXP3	0.167	***		0.175	***
	CCR8	0.237	****		0.245	****
**T cell exhaustion**	PDCD1	0.097	*		0.103	*
	CTLA4	0.146	***		0.15	**
	LAG3	0.155	***	0.15		**
	HAVCR2	0.152	***	0.156		***
	GZMB	0.089	*	0.075		0.109
**CAF**	FAP	0.217	****	0.185		****
	PDGFRα	0.178	****	0.15		**
	PDGFRβ	0.11	*	0.082		*
**MDSC**	CD11b	0.189	****	0.172		***
	Cd33	0.155	***	0.149		**
**PMN-MDSC**	CD15	0.326	****	0.33		****
**M-MDSC**	CD14	0.124	**	0.112		*

*Abbreviations*: *TAM* Tumor associated macrophage, *Treg* Regulatory cell, *CAF* Cancer-associated fibroblast, *MDSC* myeloid-derived suppressor cell, *PMN-MDSC* Polymorphonuclear myeloid-derived suppressor cell, *M-MDSC* Monocytic myeloid-derived suppressor cell, *Cor*., R value of Spearman’s correlation

^*^P < 0.05

^**^P < 0.01

^***^P < 0.001

^****^P < 0.0001

### Result 7: TOPK-mediated immunosuppressive TME formation

**Fig. 6:**
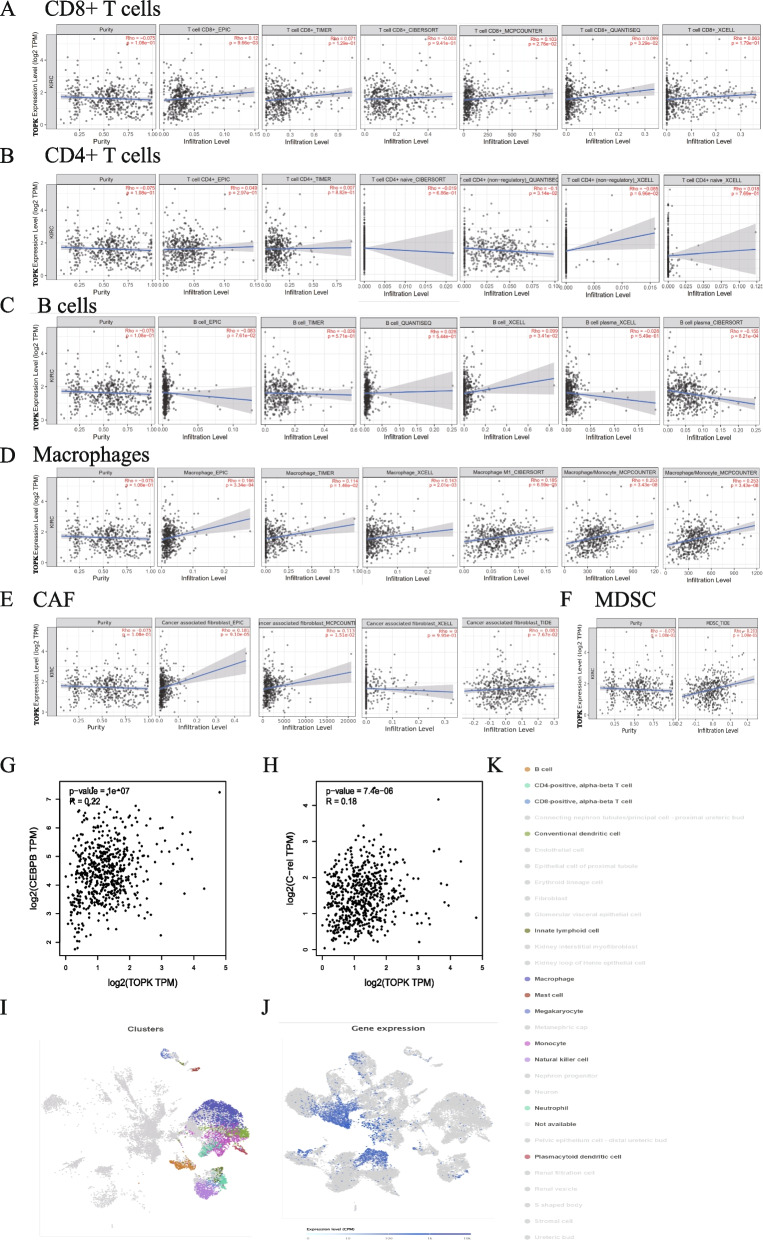
TOPK is associated with increased infiltration of immunosuppressive cells and reduced presence of immune-active cells in KIRC. **A**–**F** Immune cell infiltration analysis shows associations between TOPK expression and infiltration levels of CD8⁺ T cells, CD4⁺ T cells, B cells, macrophages, cancer-associated fibroblasts (CAF), and myeloid-derived suppressor cells (MDSCs). **G**–**H** TOPK expression is positively correlated with inflammatory transcription factors CEBPB and C-Rel. **I**–**K** Single-cell analysis of TOPK expression across different immune cell types in KIRC

To validate the influence of TOPK expression on the TME, we conducted additional investigations. After adjusting for tumor purity, the analysis revealed that immune-active cells, specifically B cells and CD4 + T cells, exhibited a negative correlation with TOPK expression. In contrast, immune-inhibitory cells, such as CAF and MDSC, displayed a positive correlation with TOPK expression (Fig. 6A-F). Furthermore, we also observed a significant positive correlation between TOPK and the expression of genes associated with the expansion of MDSC, including C/EBPb and c-Rel (Fig. 6G, H).

To further elucidate the relationship between TOPK and the infiltration of tumor cells within the KIRC, we assessed the single-cell sequencing data from the phs002065.v1.p1 dataset using the U-MAP method and observed that TOPK is correlated with various immune cell types, including CD8 + T cells, helper T cells, tp1, tp2, NK cells, B cells and TAMs (Fig. 6I-K); The GSE121636 dataset containing 3 KIRC samples and 3 control samples was further enrolled. After data processing, the cell clusters were annotated by UMAP plot, as presented in Fig. S2A. Single-cell resolution analysis revealed the expression pattern of TOPK in KIRC, visualized through UMAP dimensionality reduction and violin plots (Fig. S2B-D). The results indicated that TOPK could be over-expressed in Tm-Ki67 cells.Meanwhile, we further analyzed and found that TOPK has a positive correlation with several immunosuppressive factors such as CCL3, CCL4, CXCL2, etc. (Fig. S3).

### Result: 8 TOPK KO mice had smaller tumors

**Fig. 7:**
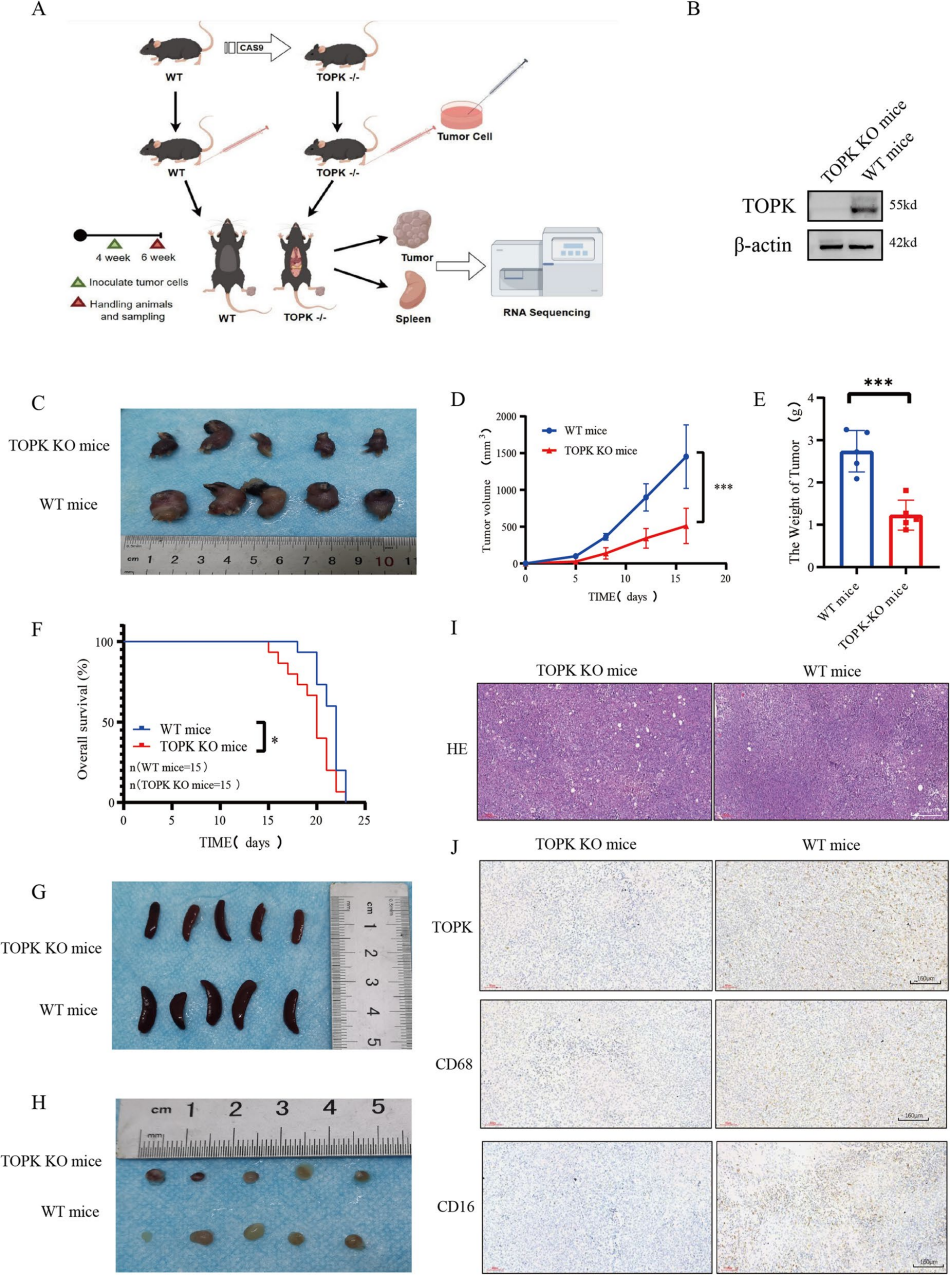
Experimental validation of TOPK KO mice model. **A** Flowchart for animal experiments. **B** Western blot analysis of TOPK expression in the testicular tissues of WT mice and TOPK knockout mice. **C**-**E** Representative images of tumors (**C**) and tumor volume (**D**), and weight (**E**) for two groups of mice (*n* = 5). **F** Survival time of mice in two groups with tumors (*n* = 5). **G** Representative images of spleens for two groups of mice (*n* = 5). **H** Representative images of lymph nodes for two groups of mice (*n* = 5). **I** HE staining of mouse tumor tissues. Scar bar = 200 μm. **J** IHC staining of mouse tumor tissues and quantification of positive cell rates. Scar bar = 160 μm. *p < 0.05; **p < 0.01; ***p < 0.001

To gain deeper insights into TOPK's impact on immune function, we used a TOPK knockout mouse model (Fig. 7A). Considering that it was previously reported that TOPK is predominantly expressed in normal human testicular tissue, with limited expression in other tissues [20], we confirmed reduced TOPK expression in testicular tissue from both wild-type (WT) and homozygous knockout (KO) mice (Fig. 7B). Importantly, TOPK KO mice were viable, fertile and exhibited no apparent phenotypic abnormalities compared to their WT littermates.

Due to the fact that approximately 20–30% of kidney cancer patients already have metastasis at diagnosis, with lymph node metastasis being one of the common forms of metastasis, this study aims to discuss the impact of TOPK on kidney cancer and lymph node metastasis. Next, we implanted tumor cells into the footpads of both WT and homozygous KO mice and found that the TOPK KO mice had smaller volume (Fig. 7C, D) and lighter weight (Fig. 7E) tumors and demonstrated prolonged survival (Fig. 7F). Further examination revealed enlarged spleens in TOPK KO mice (Fig. 7G), with no substantial differences in popliteal lymph nodes (Fig. 7H).

Tumor tissues from mice were sectioned, and HE staining confirmed the presence of tumor tissue (Fig. 7I). Subsequent IHC staining analysis demonstrated significantly reduced TOPK expression in TOPK-KO mice compared to wild-type counterparts (Fig. 7J). The proportion of CD68-positive macrophages and CD16-positive NK cells (Fig. 7J) was also markedly decreased, providing compelling evidence that TOPK deficiency substantially inhibits infiltration and activity of macrophages and NK cells within tumor tissues.

### Result 9: TOPK is closely related to the inflammatory pathway

**Fig. 8:**
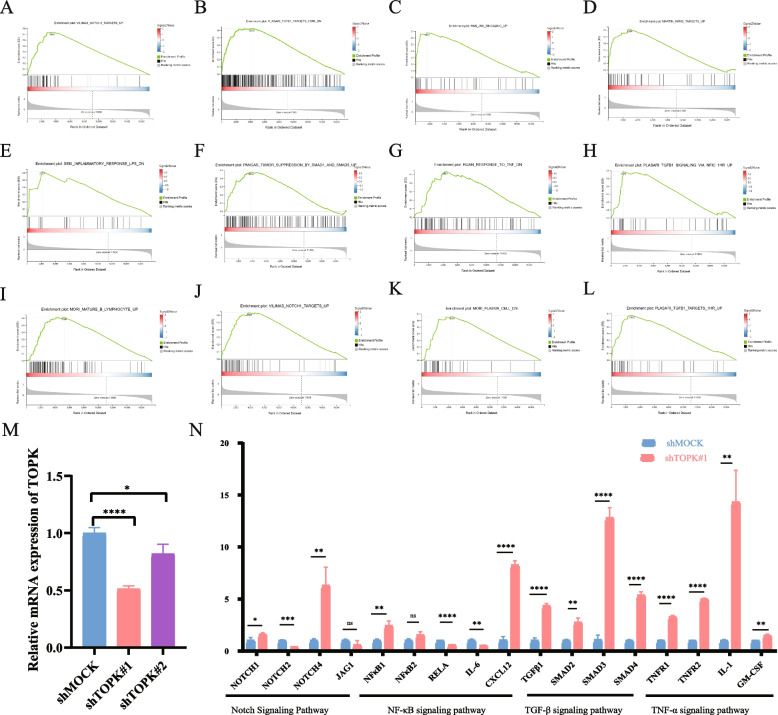
GSEA enrichment analysis and RT-qPCR revealed the TOPK-KO mice hallmarks associated with TOPK in KIRC. **A**-**L** Gene set enrichment analysis (GSEA) indicating that tumor hallmarks were enriched in TOPK KO mice. **M** Knock down TOPK in the 786-O cell line and verify using RT qPCR. **N** RT-qPCR validating that Notch signaling pathway, NF-κB signaling pathway, TGF-β signaling pathway and TNF-α were enriched in TOPK KO mice.*p < 0.05; **p < 0.01; ***p < 0.001

Subsequently, we conducted RNA-seq analysis on mouse spleen and tumor tissues to gain insights into the function of TOPK. We subjected the spleens and tumors of the above mouse models to high-throughput transcriptome sequencing and conducted Gene Set Enrichment Analysis (GSEA) using the clusterprofiler package on the obtained data. The results revealed that following TOPK knockout, several pathways, including NOTCH1, TGF-β1, JNK, NF-KB, Inflammatory, Samd1/smad5, TNF, B cell mature, Notch1, Plasma cell, were upregulated (Fig. 8A-L). Notably, most of these pathways were clustered in inflammation-related pathways, highlighting the close connection between TOPK and inflammation pathways.

Furthermore, we established a TOPK-knockdown 786-O cell line (Fig. 8M) and used PCR to validate key genes in the NOTCH signaling pathway, NF-KB signaling pathway, TNF-α signaling pathway, and TGF-β signaling pathway (Supplemental Table 1), which indicated that after TOPK knockdown, the expression levels of pivotal genes such as NOTCH1, NOTCH4, NF-KB1, CXCL12, TGF-β, SMAD2/3/4, TNFR1/2, IL-1 and GM-CSF were significantly increased (Fig. 8N). Given the intricate relationship between inflammation signaling and the tumor immune microenvironment, these results suggest a close association between TOPK and inflammation pathways. Hence, the potential mechanisms underlying the immunosuppressive TME related to TOPK are likely intertwined with inflammation signaling pathways.

### Result 10: Correlation between TOPK and treatments of KIRC

**Fig. 9:**
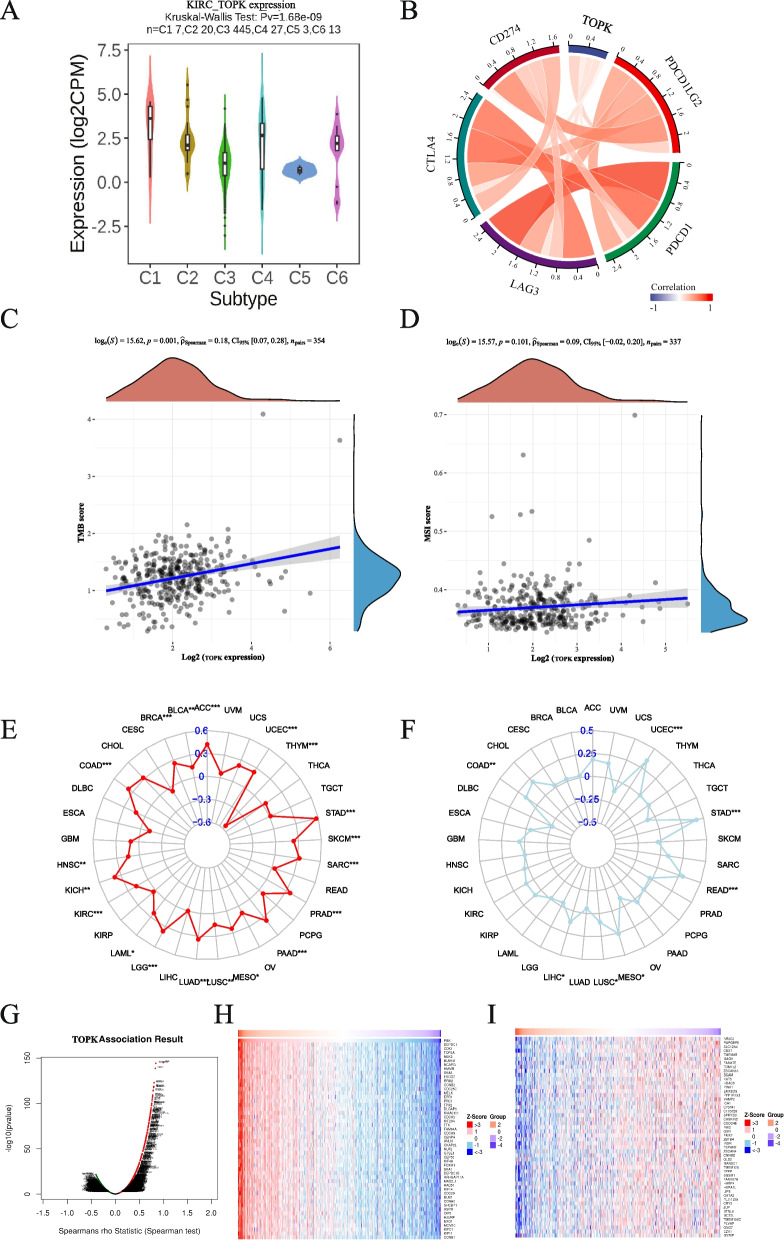
Immune therapy in TOPK. **A** Correlation of TOPK expression and immune subtype. **B** correlation of TOPK expression and immune checkpoint (CD274, CTLA4, LAG3, PDCD1, PDCD1LG2). **C**, **D** Correlation of TOPK expression with TMB and MSI in KIRC. **E**, **F** Correlation of TOPK expression with TMB and MSI in pan-cancer. **G** Volcanic plot pf co-expressed profiling of TOPK in KIRC via the Linked Omics data base. **H**, **I** Heatmaps of the 50 positively(H) and 50 negatively. **I** Related genes with TOPK are displayed. *p < 0.05; **p < 0.01; ***p < 0.001

Furthermore, we investigated the relationship between TOPK expression and pan-cancer immune subtypes, which encompass six categories: Cl (Wound Healing), C2 (IFN-γ Dominant), C3 (Inflammatory), C4 (Lymphocyte Depleted), C5 (Immunologically Quiet), and C6 (TGF-β Dominant) [21] (Fig. S4). In KIRC, we observed that TOPK exhibited the highest expression in C4 and C6 (Fig. 9A). We also examined the association between TOPK and crucial immune checkpoint genes in KIRC, including CD274 (PD-L1), CTLA4, PDCD1LG2 (PD-L2), LAG3, and PDCD1 (PD-1). As illustrated in Fig. 9B, the expression levels of TOPK were positively correlated with these immune checkpoint genes, suggesting that inhibitors of TOPK have great potential in combination with immunotherapy.

Tumor mutational burden (TMB) and microsatellite instability (MSI) are emerging biomarkers that serve as indicators of the effectiveness of immunotherapy and have been linked to clinical treatment outcomes and prognosis [22]. Thus, we explored the association between TOPK expression levels and TMB, as well as MSI, which revealed a positive correlation between TOPK expression and TMB in KIRC, while its association with MSI was not statistically significant (Fig. 9C-F).

### Result 11: TOPK expression and potential drug targets for KIRC

To explore potential associations between TOPK and drug treatment targets in KIRC, we constructed a co-expression network for TOPK in KIRC (Fig. 9G) and used positive and negative heatmaps to visually represent the top 50 genes exhibiting positive and negative correlations with TOPK, respectively (Fig. 9H, I).

Next, we used the CMap database to predict potential drug targets for KIRC using the top 50 genes positively and negatively correlated with TOPK, which were then ranked using a scoring system. We also identified the top 20 drugs/molecules that were positively and negatively correlated (Table 3, 4). Among these, compounds such as the tyrosine kinase inhibitor Tandutinib [23], the cell cycle inhibitor Palbociclib [24, 25], the anti-angiogenic agent Brivanib [26, 27], the anti-apoptotic B-cell lymphoma 2 (Bcl-2) inhibitor Navitoclax [28, 29], the CDK inhibitor Purvalanol A [30] and the histone deacetylase inhibitor Vorinostat [31] were validated as effective treatments for cancer, with their corresponding scores indicating their potential efficacy. Additionally, other drugs/molecules such as Sj-172550, Deferiprone, AC-55649, TG100-115, Aminopurvalanol-a, Mycophenolic Acid, Mycophenolate Mofetil, JAK3-Inhibitor-VI, Floxuridine, Methotrexate, Scriptaid, Amsacrine, Palbociclib and Zalcitabine could also be considered as promising therapeutic options for KIRC.

**Table 3:** The top 15 compounds with positive correlations were obtained from CMap

Rank	Score	Type	Name	Description	Target
2	99.89	cp	triacsin-c	Adrenergic receptor antagonist	ACSL1
4	99.86	cp	valproic-acid	HDAC inhibitor	ABAT, HDAC1, SCN1A, SCN3A, ACADSB, ALDH5A1, HDAC2, HDAC9, OGDH, SCN10A, SCN11A, SCN1B, SCN2A, SCN2B, SCN3B, SCN4A, SCN4B, SCN5A, SCN7A, SCN8A, SCN9A
7	99.68	cp	tyrphostin-AG-112	Protein tyrosine kinase inhibitor	EGFR
8	99.68	cp	SJ-172550	MDM inhibitor	MDM4
9	99.65	cp	HO-013	PPAR receptor agonist	PPARG
11	99.61	cp	navitoclax	BCL inhibitor	BCL2, BCL2L1, BCL2L2
12	99.54	cp	PT-630	Dipeptidyl peptidase inhibitor	DPP4, FAP
13	99.52	cp	deferiprone	Chelating agent	CYP4F2
15	99.4	cp	tandutinib	FLT3 inhibitor	FLT3, KIT, PDGFRA, PDGFRB, CSF1R, PDGFD
16	99.4	cp	doxercalciferol	Vitamin D receptor agonist	VDR
18	99.3	cp	GANT-58	GLI antagonist	DHH, GLI1, IHH
19	99.3	cp	AC-55649	Retinoid receptor agonist	RARB, RARA
22	99.19	cp	TG100-115	−666	PIK3CG, PIK3CA, PIK3CB, PIK3CD
23	99.19	cp	AR-C133057XX	Nitric oxide synthase inhibitor	NOS2, NOS1
24	99.15	cp	brivanib	FGFR inhibitor	FGFR1, KDR, FLT1, CYP3A4, FGFR2, FGFR3, FLT4, KCNH2
25	99.15	cp	BAY-K8644	Calcium channel activator	CACNA1C
26	99.14	cp	zalcitabine	Nucleoside reverse transcriptase inhibitor	/
28	99.01	cp	iloperidone	Dopamine receptor antagonist	DRD2, HTR2A, HTR1A, HTR6, HTR7, ADRA1A, ADRA2C, DRD1, DRD3, DRD4, HRH1
29	98.94	cp	maraviroc	CC chemokine receptor antagonist	CCR5, CYP3A5
30	98.91	cp	NF-449	Purinergic receptor antagonist	P2RX1

**Table 4:** The top 15 compounds with negative correlations were obtained from CMap

Rank	Score	Type	Name	Description	Target
8542	−99.65	cp	aminopurvalanol-a	Tyrosine kinase inhibitor	CDK1, CDK2, CDK5, CDK6
8532	−99.47	cp	purvalanol-a	CDK inhibitor	CDK1, CDK2, CDK4, CDK5, CCND1, CCNE1, CSNK1G3, RPS6KA1, SRC
8524	−99.22	cp	HG-5–113-01	Protein kinase inhibitor	ABL1, LTK, STK10
8522	−99.19	cp	mycophenolic-acid	Dehydrogenase inhibitor	IMPDH1, IMPDH2
8515	−99.12	cp	mycophenolate-mofetil	Dehydrogenase inhibitor	IMPDH1, IMPDH2, HCAR2
8516	−99.12	cp	JAK3-inhibitor-VI	JAK inhibitor	JAK3
8514	−99.05	cp	floxuridine	DNA synthesis inhibitor	TYMS
8504	−98.98	cp	teniposide	Topoisomerase inhibitor	TOP2A, CYP3A5
8496	−98.91	cp	SN-38	Topoisomerase inhibitor	TOP1
8490	−98.84	cp	THM-I-94	HDAC inhibitor	HDAC1, HDAC10, HDAC2, HDAC3, HDAC6, HDAC8
8489	−98.8	cp	ISOX	HDAC inhibitor	HDAC6
8487	−98.77	cp	NCH-51	HDAC inhibitor	HDAC1, HDAC10, HDAC11, HDAC2, HDAC3, HDAC4, HDAC5, HDAC6, HDAC7, HDAC8, HDAC9
8484	−98.73	cp	angiogenesis-inhibitor	Angiogenesis inhibitor	EGFR
8477	−98.66	cp	vorinostat	HDAC inhibitor	HDAC1, HDAC2, HDAC3, HDAC6, HDAC8, HDAC10, HDAC11, HDAC5, HDAC9
8467	−98.52	cp	vidarabine	Antiviral	ADCY5
8466	−98.48	cp	amonafide	Topoisomerase inhibitor	TOP2A, TOP2B
8460	−98.45	cp	methotrexate	Dihydrofolate reductase inhibitor	DHFR, AOX1, FOLR1, TYMS
8461	−98.45	cp	scriptaid	HDAC inhibitor	HDAC1, HDAC2, HDAC3, HDAC4, HDAC5, HDAC6, HDAC7, HDAC8, HDAC9
8462	−98.45	cp	amsacrine	Topoisomerase inhibitor	TOP2A, KCNH2
8463	−98.45	cp	palbociclib	CDK inhibitor	CDK4, CDK6, CCND3

## Discussion

KIRC poses a significant global health challenge, with a growing incidence worldwide and a mortality rate of approximately 20% [32]. While surgical interventions offer reliable prognostic benefits for early-stage patients, the survival prospects for those in advanced stages remain limited [33]. As our understanding of immunology advances, immunotherapy treatments such as immune checkpoint inhibitors (ICIs), therapeutic vaccines and adoptive cell therapy have emerged as a significant field of research in both fundamental science and clinical applications for cancer treatment. Currently, ICIs have gained approval for KIRC treatment and are undergoing preclinical investigations and clinical trials [34, 35]. Consequently, the identification of novel biomarkers for diagnosing and selecting treatments for KIRC patients holds significant importance. In this study, we aimed to elucidate the biological landscape of TOPK in KIRC, and through a series of rigorous bioinformatics analyses, we confirmed that TOPK over-expressed in KIRC patients is linked to unfavorable outcomes.

Subsequently, we sought to identify factors contributing to the elevated expression of TOPK in KIRC. Genetic mutations were initially considered, as they frequently influence gene expression and alternative splicing across the genome [15]. However, mutations in TOPK were rare and showed no functional relevance in KIRC. We next examined promoter methylation, a stable epigenetic modification known to regulate gene expression in a temporal and spatial manner [17]. Interestingly, we observed significantly elevated levels of promoter methylation in TOPK in KIRC tumors, despite concurrently increased mRNA and protein expression. While promoter methylation is conventionally associated with transcriptional repression [36, 37]. Nonetheless, numerous studies suggest that this association may not always hold, as in some cases, high promoter methylation has been linked to increased transcriptional activity [38], as observed in genes such as AWT1 [39], BIRC5 [40], EBF3 [41], FAX2 [42], PAX2 [43], PDPN [44] and TERT [45], all of which have been found to exhibit high transcriptional activity despite high promoter methylation. These paradoxes may arise due to the methylation occurring at non-critical CpG sites, methylation-mediated blockage of repressive transcription factors, or recruitment of activating chromatin complexes. Additionally, gene expression is governed by multiple epigenetic layers—including histone modifications, chromatin accessibility, transcription factor activity, and non-coding RNAs—which may collectively override canonical methylation effects. Therefore, the observed co-occurrence of high promoter methylation and elevated TOPK expression in KIRC may reflect a noncanonical regulatory mechanism. This atypical relationship warrants further mechanistic investigation using functional epigenomic assays (e.g., ChIP-seq, bisulfite sequencing) to delineate the precise interplay between methylation and TOPK transcriptional control.

Numerous studies have demonstrated the involvement of TOPK in promoting the progression of various cancers. For instance, in gastric cancer, FYN directly interacts with TOPK in gastric cancer cells, leading to the phosphorylation of TOPK at Y272 and subsequent increases in cell proliferation and metastasis [10]. In esophageal cancer, TOPK enhances the YB1/eEF1A1 signaling pathway, facilitating tumor growth both in vitro and in vivo. In prostate cancer, TOPK promotes the expression of the androgen receptor splice variant (ARv7), driving androgen-independent growth and regulating tumor-specific radio-sensitivity [46, 47]. Additionally, the interactions between TOPK and ERK have been implicated in the progression of various tumors, including colorectal cancer [48], non-small cell lung cancer [49] and esophageal cancer [50]. However, most of these studies have primarily focused on intracellular aspects of tumor cells, and the role of TOPK as a protein kinase in the tumor immune microenvironment remains inadequately explored.

Several studies have provided insights into TOPK's role in various contexts, including cerebral ischemia–reperfusion injury, acute kidney injury (AKI) to chronic kidney disease (CKD transition), and colorectal cancer. In cerebral ischemia–reperfusion injury, TOPK promotes the polarization of macrophages towards the M2 phenotype, exerting a neuroprotective effect by positively regulating M2 polarization of microglial cells/macrophages through the inhibition of HDAC1/HDAC2 activity [51]. In the transition from acute kidney injury (AKI) to chronic kidney disease (CKD), the SGK3/TOPK signaling pathway has been shown to exhibit a counteractive effect, promoting fibrosis in renal tubular epithelial cells and CD206 M2 macrophage polarization [52]. In colorectal cancer, TOPK has been suggested as a favorable prognostic biomarker related to anti-tumor immunity [53], although further validation through basic experiments is necessary. We have also uncovered that TOPK in kidney cancer enhances the proliferation and migration of KIRC cells by upregulating the expression of PD-L1, thereby mediating immune evasion in KIRC [14]. These findings highlight a significant connection between TOPK and the immunosuppressive microenvironment of tumors, which led us to further investigate TOPK's role in the tumor immune microenvironment of kidney cancer, which showed that high TOPK expression in KIRC was closely associated with the infiltration of immunosuppressive cells and a deficiency in immune-active cells within the immune microenvironment. These results are in line with previous findings, suggesting that in KIRC, TOPK may promote the development of immunosuppressive TME.

In this present study, we report a robust correlation between TOPK overexpression and the infiltration of immunosuppressive cells, particularly MDSCs and CAFs. These CAFs play a crucial role in tumor progression and drug resistance by engaging in various mechanisms, including fibrosis, angiogenesis, immune regulation and cancer metabolism. They facilitate tumor cells in evading immune surveillance through the reduction of immune cell infiltration, suppression of immune cell activation, and secretion of immunosuppressive molecules [54]. MDSCs, on the other hand, are a pivotal subset of immunosuppressive cells, and accumulating evidence highlights their significant role in driving the immunosuppressive TME [55]. MDSCs can hinder T cell activation and function, reduce natural killer (NK) cell activities, and impede the antigen presentation process, thus suppressing the immune system to facilitate tumor escape and growth [56]. Both C/EBPb and c-Rel have been implicated in MDSC expansion [57]. Further investigation revealed a significant positive correlation between C/EBPb, c-Rel and TOPK expression, providing support to the idea that TOPK signaling may contribute to the accumulation of functional MDSCs. Furthermore, single-cell sequencing data have demonstrated that TOPK is associated with various immune cell types, including CD8 + T cells, helper T cells, tp1, tp2, NK cells, B cells, TAM cells, and Tm Ki-67 cells. Notably, TOPK expression is particularly elevated in proliferating memory T cells. This observation may be attributed to the role of memory T cells in long-term survival and rapid response to reinfection. However, sustained activation of TOPK could potentially induce an exhaustion phenotype in T cells, leading to a loss of effector function. Such cells may enter a state of"proliferative exhaustion,"where they can initially expand but ultimately lose their anti-tumor efficacy. Additionally, this may result in memory T cells adopting a"tumor-friendly"or"pseudo-memory"phenotype, appearing phenotypically similar to functional memory T cells while being dysfunctional or even tumor-promoting. This aligns with the role of TOPK in facilitating tumor progression, as supported by previous studies. Nonetheless, the precise mechanisms underlying these observations require further experimental validation.Therefore, we conclude that TOPK serves as an effective biomarker for shaping the tumor immune microenvironment in KIRC.

Following this, we used a TOPK KO mouse model and found that the removal of TOPK significantly suppressed tumor growth and prolonged the survival of mice carrying these tumors, which highlight the pivotal oncogenic role played by TOPK. Next, we conducted high-throughput transcriptome sequencing on the tumors derived from this mouse model, with the results of bioinformatics enrichment analysis indicating a close relationship between TOPK and inflammatory-related pathways, which predominantly included the NOTCH, NF-KB, TNF-α and TGF-β signaling pathways. The interplay between inflammatory signaling pathways and the tumor immune microenvironment is intricate and not yet comprehensively understood [58]. Inflammation can either promote immune cell infiltration and activation or lead to immune evasion and resistance [59], with this complexity potentially tied to the spatiotemporal dynamics of tumor progression. It should be noted that the TOPK knockout animal model offered valuable insights into how other genes respond to the presence or absence of TOPK. However, it should also be acknowledged that the biological information gathered differs from previous data obtained from tumors in mice with subcutaneously implanted TOPK-KD Renca cells. Consequently, further research at the animal or cellular level is required to validate our findings.

Collectively, these findings suggest that the overexpression of TOPK is potentially linked to the infiltration of immunosuppressive cells and a shortage of immune-active cells, which may contribute to the formation of an immunosuppressive TME. The complex role of TOPK in cancer prognosis—shaped by environmental cues—highlights its dual function in tumor progression and immune regulation, supporting its potential as a therapeutic target. Although multiple structurally distinct TOPK inhibitors have been assessed in preclinical and clinical studies, none have yet received regulatory approval. In our previous work, we linked TOPK phosphorylation to sorafenib resistance, underscoring its therapeutic relevance. In the present study, bioinformatics analysis (CMap) identified several candidate compounds targeting TOPK-associated signatures in KIRC, most notably tyrosine kinase inhibitors such as Tandutinib and Palbociclib.

Notably, several of these compounds may exert their effects by modulating immune-related pathways. Tandutinib is a multi-kinase inhibitor that targets FLT3 and PDGFR signaling, both of which are implicated in immune suppression and tumor–stroma interactions. Palbociclib, a CDK4/6 inhibitor, has been shown to enhance anti-tumor immunity by promoting T cell infiltration and reducing Treg accumulation. The convergence of these drugs on immune-regulatory pathways aligns well with our findings that TOPK is involved in establishing an immunosuppressive tumor microenvironment, suggesting a potential therapeutic synergy. These candidates may thus represent promising leads for targeting TOPK-mediated immune modulation in KIRC. Collectively suggesting that the overexpression of TOPK is potentially linked to the infiltration of immunosuppressive cells and a shortage of immune-active cells, which may contribute to the formation of an immunosuppressive TME.

However, our study, while maintaining rigorous analysis and high-quality experiments, possessed certain limitations. Firstly, the utilization of multiple databases in our analysis may have introduced systemic biases, potentially affecting the robustness of our conclusions. Secondly, although this study primarily focuses on KIRC, we used RM-1 cells in C57BL/6 mice to construct an immunocompetent tumor model for in vivo validation. RM-1, a murine prostate cancer cell line, was chosen due to its compatibility with the C57BL/6 genetic background and its robust tumorigenicity, which enabled effective assessment of immune modulation upon TOPK deficiency. We acknowledge that RM-1 is not of renal origin, and this limits the model’s specificity for KIRC. However, this approach provided valuable insights into the immunosuppressive effects of TOPK in a physiologically relevant immune environment. In future studies, we plan to adopt the RENCA cell line in BALB/c mice to establish a more accurate syngeneic model for renal cancer. Lastly, the mechanistic pathways by which TOPK drives an immunosuppressive tumor microenvironment remain incompletely understood and warrant further mechanistic investigations.

## Conclusion

In summary, our findings demonstrate that TOPK upregulation is closely associated with poor prognosis and altered immune infiltration within the tumor microenvironment (TME) in KIRC. These results support TOPK as a promising target for therapeutic intervention and a potential immunotherapeutic strategy in renal cancer. While our in vivo findings were based on a non-renal tumor model, they nonetheless provide valuable insights into the immunoregulatory role of TOPK and warrant further validation using kidney-specific systems.

## Materials and methods

### Data collection and expression analysis

The RNA-seq data for both pan-cancer and TCGA-KIRC were obtained from the UCSC Xena browser (https://xenabrowser.net) [60]. This comprehensive dataset consisted of gene expression profiles from 28 normal tissue samples extracted from the GETx database and an additional 72 adjacent normal tissue samples [61]. Additionally, it encompassed 531 tumor tissue samples, along with associated clinical information and matched normal samples. To prepare the data for analysis, we performed a series of preprocessing steps using the'limma'and'affy'packages in the R programming language, which included background correction, probe ID annotation, missing value imputation, and data normalization. The'ggplot2'package was used for data visualization. Comparative analysis of KIRC patient expression was performed using the Wilcoxon rank-sum test. In addition, the TOPK protein expression data were investigated using the Clinical Proteomic Tumor Analysis Consortium (CPTAC) tool [62]. Tables 1 provide the baseline characteristics of the patients included in the analysis. These patients were categorized into high and low expression groups based on the median expression level of the TOPK gene. Subsequently, the'limma'package in the R environment was employed to perform differential gene expression analysis, utilizing specific parameter settings.

### Methylation analysis

A comprehensive analysis of the interactions between TOPK expression, genetic modifications and DNA methylation was performed. For an in-depth exploration of the promoter methylation levels of TOPK and their relevance to clinical-pathological characteristics, data from the UCLCAN database (https://ualcan.path.uab.edu/) were retrieved and assessed [63]. Additionally, the MethSurv database (https://biit.cs.ut) was used to investigate the associations between various CpG islands and their influence on TOPK expression and patient prognosis [64].

### Mutation analysis

The cBioPortal database (https://www.cbioportal.org/) is a user-friendly and powerful resource for exploring and analyzing diverse cancer genomics data, covering epigenetics, gene expression profiles and proteomics data [65, 66]. Herein, we utilized cBioPortal to assess the alterations in TOPK across 38 datasets, which collectively included 26,390 pan-cancer samples.

### Prognostic analysis

We aligned the data obtained from TCGA with the corresponding clinical information and utilized the'survival'package to conduct proportional hazard assumption tests and fit survival regression models to evaluate the prognostic significance of TOPK in KIRC patients, offering a thorough analysis of TOPK's prognostic relevance for this patient cohort.

### Functional enrichment analysis

We performed Gene Ontology (GO) and KEGG pathway enrichment analyses on the obtained differentially expressed genes (DEGs) using the'ClusterProfiler'package in R. Additionally, we conducted gene set enrichment analysis (GSEA) to explore potential biological distinctions between the two groups. Our analysis employed a comprehensive set of reference gene collections, including Hallmark gene sets. To enhance the interpretability of our results, we used the'ggridges'package for visualization.

### Immunological correlation analysis

To investigate the relationship between immune infiltration and TOPK, we calculated the StromalScore, ImmuneScore and ESTIMATEScore of the KIRC patient samples. We also evaluated the correlation between TOPK expression and immune infiltration using Spearman correlation analysis. Furthermore, we quantified the immune microenvironment utilizing the Timer 2.0 database (cistrome.org) [67], using various algorithms such as XCELL [68], MCPCOUNTER [69], CIBERSORT [70], TIMER [71], EPIC [72], and QUANTISEQ [73]. Moreover, Spearman correlation analysis was performed to examine the potential associations between TOPK expression and the levels of immune checkpoint markers, and the immune subtype analysis of TOPK was conducted using the TISIDB database (http://cis.hku.hk/TISIDB) [74]. *P* < 0.05 was considered statistically significant.

### Acquisition and analysis of KIRC single-cell RNA sequencing (scRNA-seq) data

To conduct a comprehensive analysis of TOPK expression across various immune cell types, we performed single-cell analysis using two datasets obtained from the Single Cell Expression Atlas database and the scRNASeqDB database (https://bioinfo.uth.edu/scrnaseqdb/) [75].

The KIRC scRNA-seq data for TOPK was acquired from the GSE121636 dataset comprising 3 tumor samples and 3 control samples from the GEO online website (https://www.ncbi.nlm.nih.gov/geo/; accessed on May 2025). Thus, the measurement data were merged with the “Seurat” R package and subjected to principal component analysis [76]. Single-cell gene expression profiles were filtered to exclude cells exhibiting more than 15% mitochondrial gene content or fewer than 200 transcripts per cell, as these are considered low quality. Subsequently, the RunPCA function was applied to conduct linear dimensionality reduction through principal component analysis (PCA), and the RunUMAP function was executed to generate visual representations of the clusters. Then, the"MAESTRO"package was employed for cluster annotation, thus enabling the identification of distinct cell types [77].

### Connectivity map (CMap) analyses, TMB, and MSI

The Connectivity Map (CMap) (clue.io) is a valuable resource for rapidly identifying drugs that exhibit high correlation with a specific disease based on gene expression profile data [78]. This tool allows for the inference of potential drug mechanisms. In our investigation, the CMap database used the top 50 upregulated and downregulated genes associated with TOPK, which were sourced from The Linked Omics database (http://www.linkedomics.org/login.php), to predict potential drug targets for KIRC [79]. These potential therapeutic targets were ranked using an integrated scoring system. Tumor mutation burden (TMB) and microsatellite instability (MSI) scores were obtained from the TCGA database, and data visualization was performed using R software and the'fmsb'package.

### TOPK KO mice

Professor Zhu Feng from the Cancer Research Institute at the Affiliated Hospital of Guilin Medical University constructed the wild-type (WT) and TOPK knockout (KO) mice and kindly provided them for our study. To target the TOPK gene, CRISPR guide RNA (gRNA) sequences were designed based on the genomic structure of TOPK. The activity of these gRNA sequences was evaluated in vitro using spCas9 nuclease. Highly active gRNA targets were selected for the generation of gene-mutated mice. The most active gRNA targets were used for in vitro transcription to produce RNA, which was then microinjected into fertilized mouse eggs along with spCas9 nuclease protein. This process resulted in the creation of founder mice with genetic mutations. Sequencing near the CRISPR target sites confirmed the presence of fragment deletions in these founder mice. The first mouse with the genetic mutation (founder) was bred with a wild-type mouse to produce the F1 generation, which consisted of mutant heterozygous mice (±). DNA sequencing was performed to confirm the deletion of target gene fragments, leading to the inactivation of the target protein and, consequently, a knockout. Genotypically identical heterozygous mice (±) from the F1 generation were interbred to generate pure knockout heterozygous mice (-/-) in the F2 generation.

For the euthanasia of mice, animals were anesthetized using inhaled isoflurane at a concentration of 4% for induction and 1–2% for maintenance. Once deep anesthesia was confirmed, euthanasia was performed by cervical dislocation. This procedure was conducted following ethical guidelines to ensure a humane process while maintaining tissue integrity for further analyses.

### Cell lines and cell culture

HEK293T, RM-1 and KIRC cell line 786-O were purchased from the Chinese Academy of Sciences Cell Bank (Beijing, China). The authenticity of all cell lines was verified using Short Tandem Repeat (STR) profiling, which was conducted by the cell bank. These cells were cultured under sterile conditions at 37 °C with 5% CO_2_, using either DMEM or 1640 medium supplemented with 10% fetal bovine serum (FBS) in sterile culture flasks.

### Establishment of mouse tumor models

RM-1 cells were inoculated with (1 × 106) cells subcutaneously on the back of 6–8-week-old WT and TOPK KO mice.Tumor volumes were measured every three days along the long axis (a) and short axis (b), and tumor volumes were calculated using the following formula: V = ab2/2. Mice were sacrificed at the end of the experiment, tumors were excised and weighed, sequenced the tumor and spleen tissues of the mice.

### Chronic virus infection and stable cell line construction

Chronic viral expression vectors, which included shTOPK#2, vector control shMock, pMD2.0G, and psPAX2, were obtained from Sino Biological Co., Ltd. (Beijing, BJ). Following the manufacturer's instructions, the viral vector and packaging vectors were transfected into HEK293T cells using Lipofectamine 3000 (Invitrogen, USA). After a 6-h incubation, the culture medium was replaced, and the cells were maintained for 24 h. Subsequently, the virus particles were collected. When the cell confluence reached approximately 70–80%, 786–0 cells were infected for 24 h, followed by a change of the culture medium. The cells were then cultured for an additional 48 h and subjected to selection with puromycin (1.5 µg/mL) for a minimum of 3 days. Two shRNA sequences were designed to achieve TOPK knockdown. These sequences are as follows: 1. 5′-TTCCAGACCTTGCCCTACTAAGCTCTTCAAGAGAGAGCTTAGTAGGGCAAGGTCTGGATTTTTTC-3′; 2. 5′-TGCCTCAGAGAAGATCTCTTAGTTCAAGAGACTAAGAGATCTTCTCTGAGGCTTTTTTC-3′.

### Cell RNA extraction and RT-qPCR

Total RNA was extracted from the cells using TRIzol RNA extraction reagent (ThermoFisher). Reverse transcription for both miRNA and mRNA was conducted using RimeScript-RT-Master Mix (Takara). Subsequently, RT-qPCR was performed using the CFX96 deep-well real-time PCR detection system (BioRad) and Hieff-qPCR SYBR Green Master Mix (Yeasen). The primer sequences used are shown in the supplementary table. β-actin was used as the reference gene for data normalization.

### Protein extraction and western blotting

Cell lysis was conducted using RIPA lysis buffer (Solarbio) supplemented with protease inhibitors (Solarbio) and phosphatase inhibitors (MedChemExpress). Protein quantification was carried out using the BCA assay (Sigma-Aldrich). Subsequently, the protein lysates were loaded onto an SDS-PAGE gel and transferred onto PVDF membranes (Merck Millipore). These membranes were incubated with primary antibodies overnight at 4 °C. The primary antibodies used were TOPK (Cell Signaling Technology, Cat# 4942) and β-actin (β-actin, Cat# AC038). Following incubation with secondary antibodies, signal detection was performed using the C300 system (Azure Biosystems) with an enhanced chemiluminescence reagent (ThermoFisher).

### Statistic analysis

Statistical analyses were performed using GraphPad Prism 8.0, and all results are presented as mean values ± standard deviation (SD). Comparison between different groups was conducted using one-way analysis of variance (ANOVA), and statistical significance was established at *p* < 0.05.

## Supplementary Information

Supplementary Material 1.

Supplementary Material 2.

## Data Availability

Data that support the findings of this study are available from the corresponding author, Chen Shao, upon reasonable request.
