# Supplementary material for: TOPK promotes immune suppression in kidney renal clear cell carcinoma and emerges as a prognostic and therapeutic target
Source: BMC Cancer. 2025 Aug 18;25:1334. doi: 10.1186/s12885-025-14665-0 (PMC12359846; doi:10.1186/s12885-025-14665-0)
Supplement: Supplementary file 1 — Supplementary Material 1. [file 12885_2025_14665_MOESM1_ESM.docx]

**TOPK Promotes Immune Suppression in Kidney Renal Clear Cell Carcinoma and Emerges as a Prognostic and Therapeutic Target**

Zeyuan Zheng ^1,†^, Renhui Xiong^2,†^, Xiuyuan Sui ^1^, Liyan Li ^1^, Huimin Sun^3,*^ and Chen Shao ^1,*^

^1^ Department of Urology, Xiang’an Hospital of Xiamen University, School of Medicine, Xiamen University, Xiamen 361101, China

^2^ Department of Oncology, Xiang’an Hospital of Xiamen University, School of Medicine, Xiamen University, Xiamen 361101, China

^3^ Central Laboratory, Xiang’an Hospital of Xiamen University, School of Medicine, Xiamen University, Xiamen 361101, China

^†^ These authors contribute equally to this work.

^*^Correspondence:

Prof. Chen Shao (E-mail: cshao@xah.xmu.edu.cn)

Prof. Huimin Sun (E-mail: hmsun@xah.xmu.edu.cn)


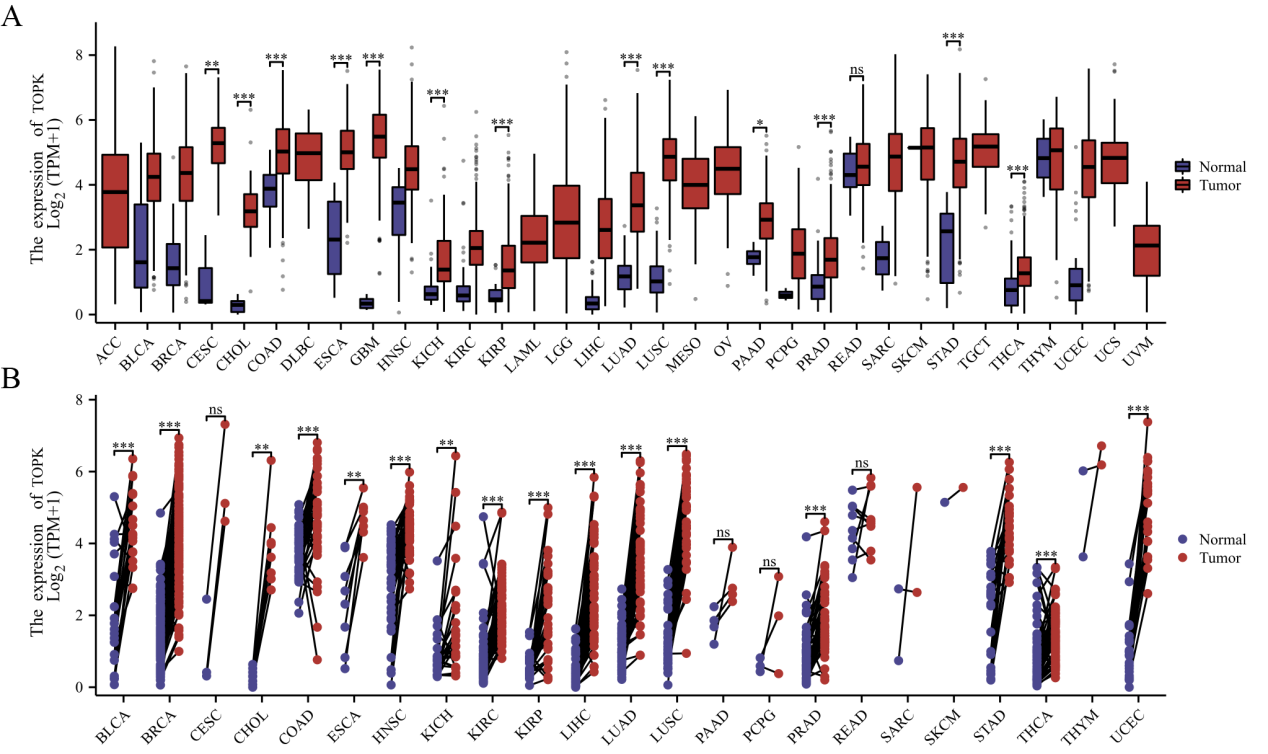


**Figure S1. TOPK expression in pan-cancer analysis.** (A)Pan-cancer analysis of TOPK expression in tumor and normal tissues in TCGA + GETx dataset. (B)Pan-cancer analysis of TOPK expression in tumor and paired normal tissues samples.*p < 0.05; **p < 0.01; ***p < 0.001.


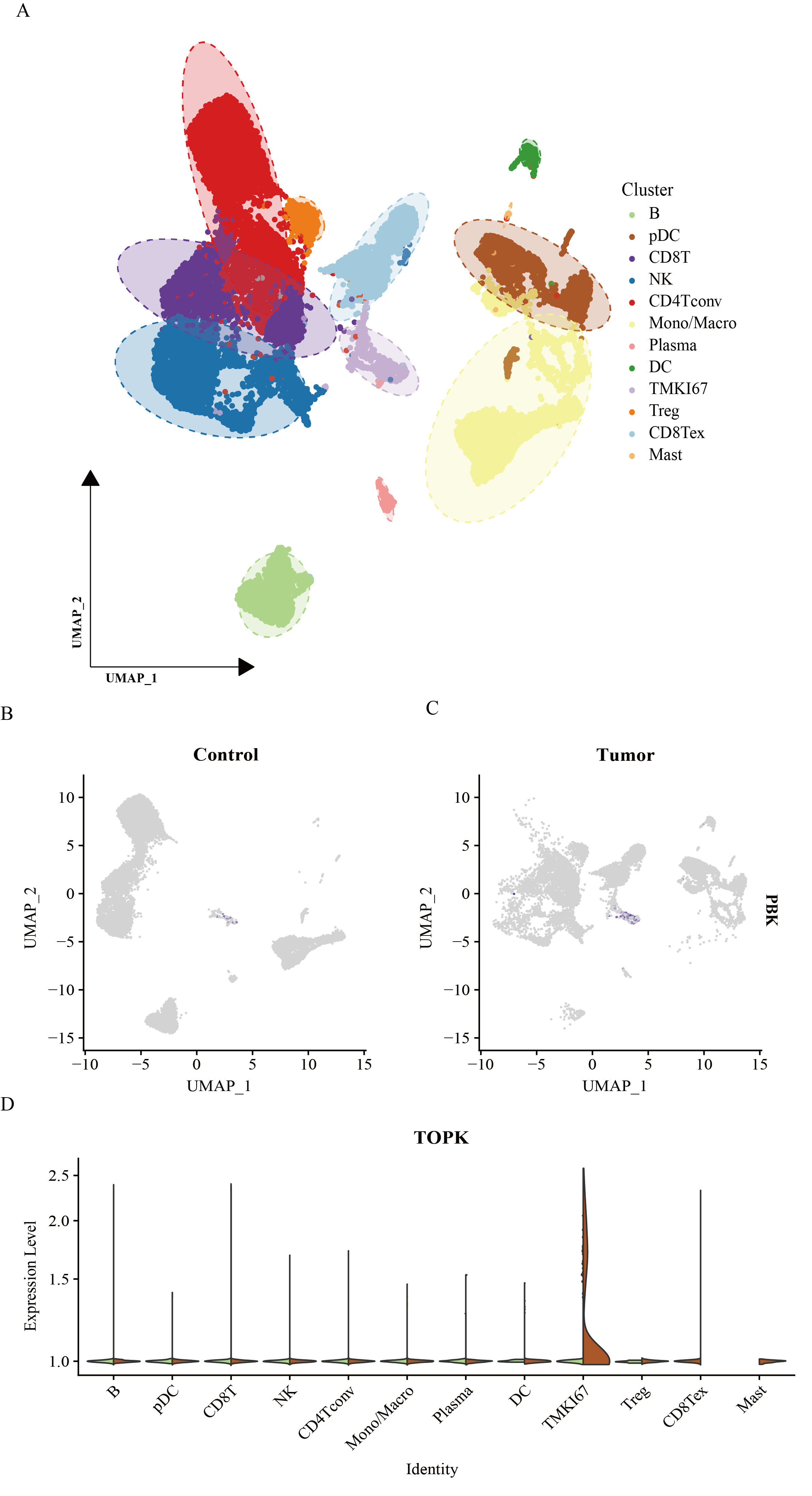


**Figure S2. GSE121636 scRNA-seq data of TOPK expression in KIRC.** (A) KIRC samples were annotated with twelve immune cell clusters by UMAP plot; (B-C) The expression of TOPK in KIRC at single-cell levels by UMAP plot; (D) TOPK had relatively higher expressions in TmKi-67 cells.


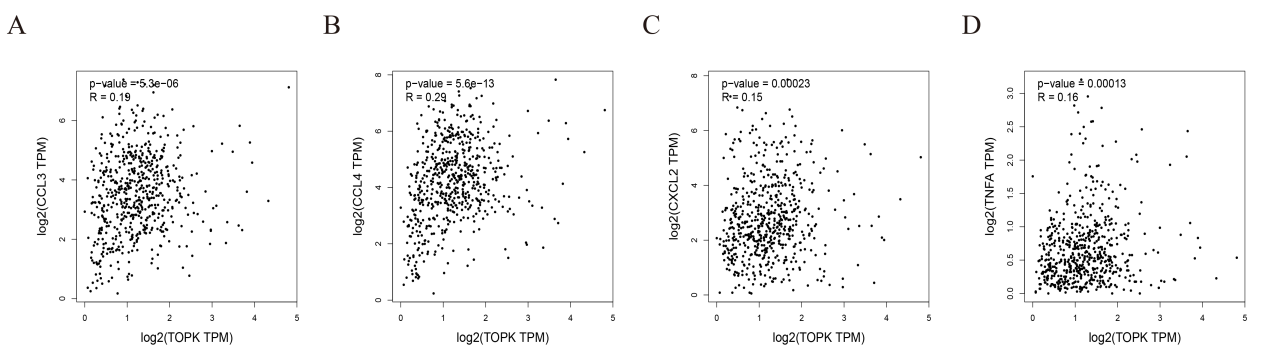


**Figure S3. Correlation of Immunosuppressive chemokines and TOPK expression.** (A-D) Correlation of CCL3 (A), CCL4 (B), CXCL2 (C), TNF-α (D) and TOPK expression.


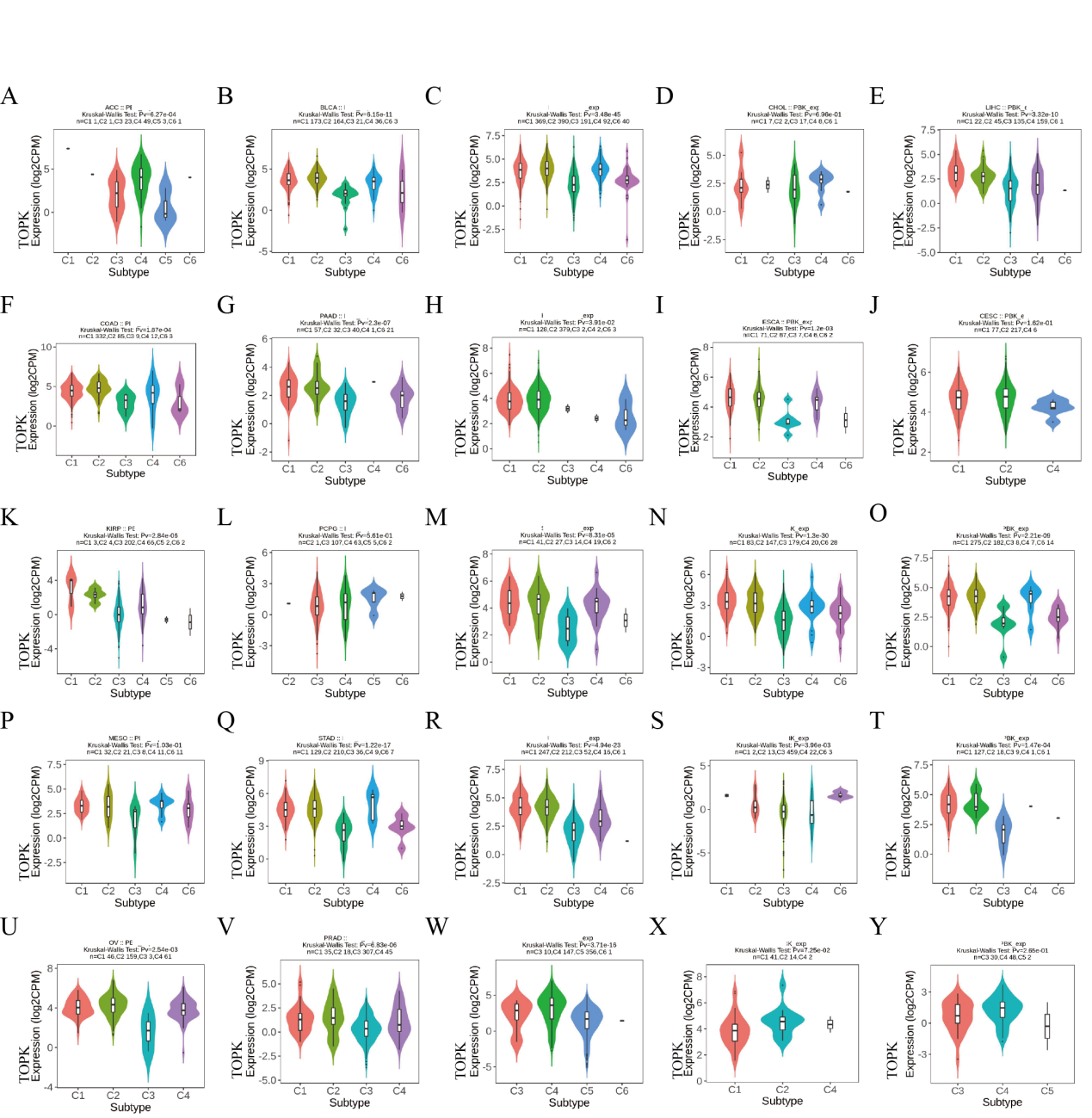


**Figure S4. Correlation of TOPK expression and immune subtype in pan-cacner.** (A) ACC (B) BLCA (C) BRCA (D) CHOL (E) LIHC (F) COAD (G) PAAD (H) HNSC (I) ESCA (J) CESC (K) KIRP (L) PCGC (M) SKCM (N) LUAD (O) LUSC (P) MESO (Q) STAD (R) UCEC (S) THCA (T) READ (U) OV (V) PRAD (W) LGG (X) UCS (Y) UVM
