# Supplementary material for: TOPK promotes immune suppression in kidney renal clear cell carcinoma and emerges as a prognostic and therapeutic target
Source: BMC Cancer. 2025 Aug 18;25:1334. doi: 10.1186/s12885-025-14665-0 (PMC12359846; doi:10.1186/s12885-025-14665-0)
Supplement: Supplementary file 2 — Supplementary Material 2. [file 12885_2025_14665_MOESM2_ESM.pdf]

Fig-7B β-actin

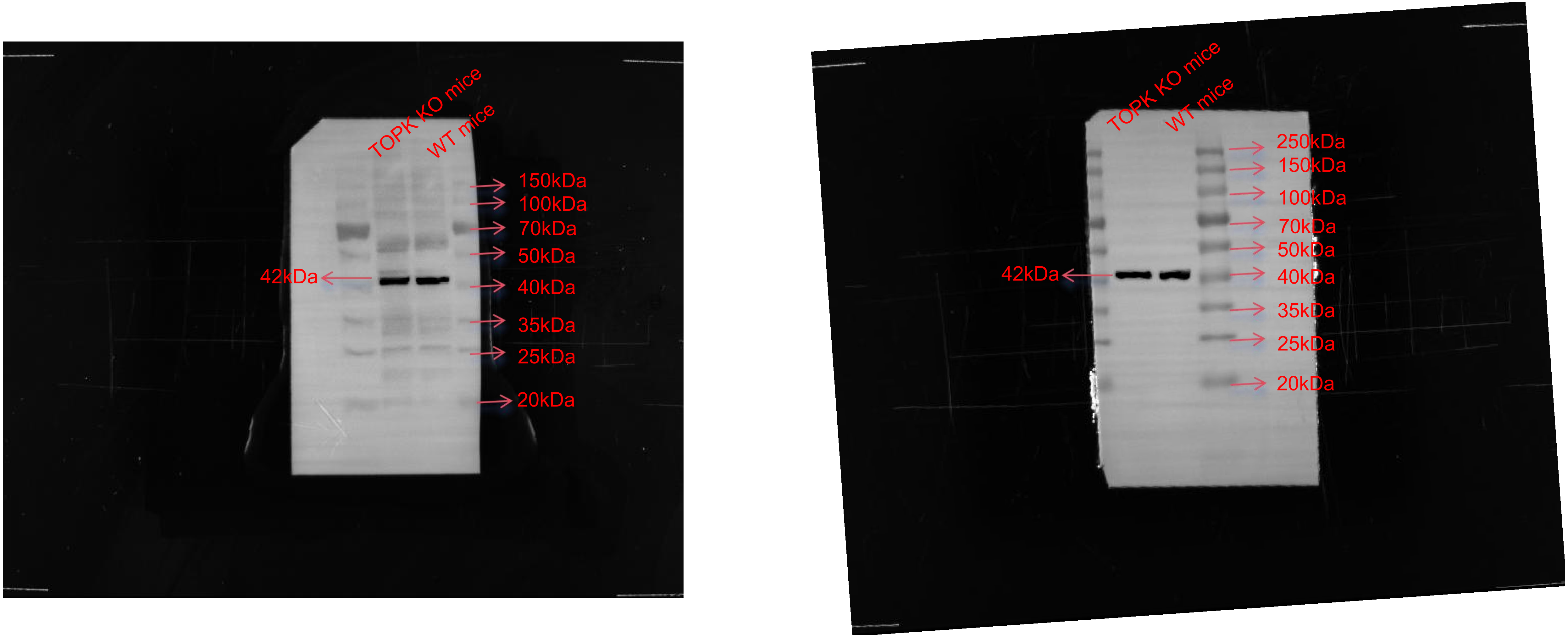

Fuller-length, original, and unprocessed Westem blot images of Anti- β-actin antibody to confim specific detection of the target antigen.(Predicted band size: 42 kDa Observed band size: 42 kDa)

Fig-7B β-actin

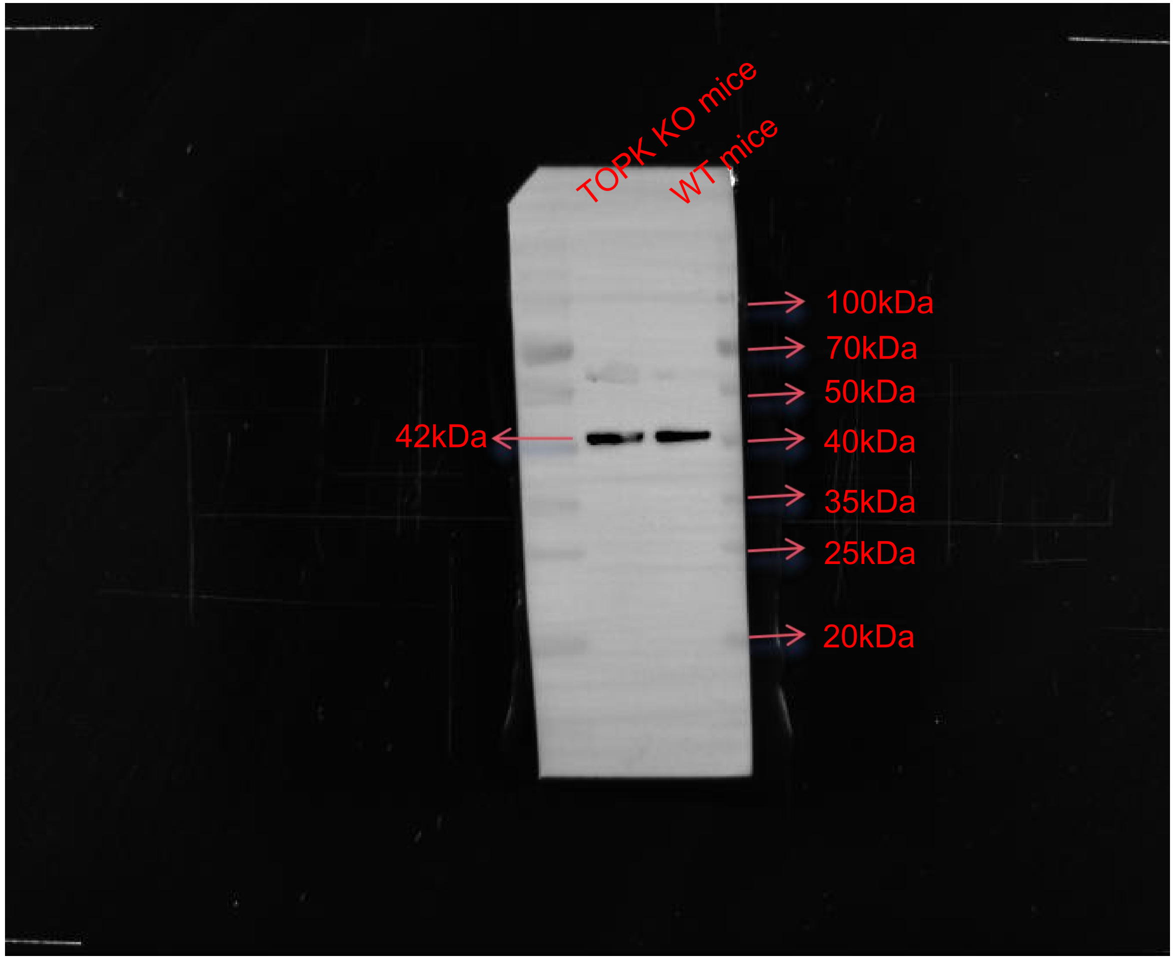

Fuller-length, original, and unprocessed Westem blot images of Anti- β-actin antibody to confim specific detection of the target antigen.(Predicted band size: 42k Da Observed band size: 42 kDa)

Fig-7B TOPK

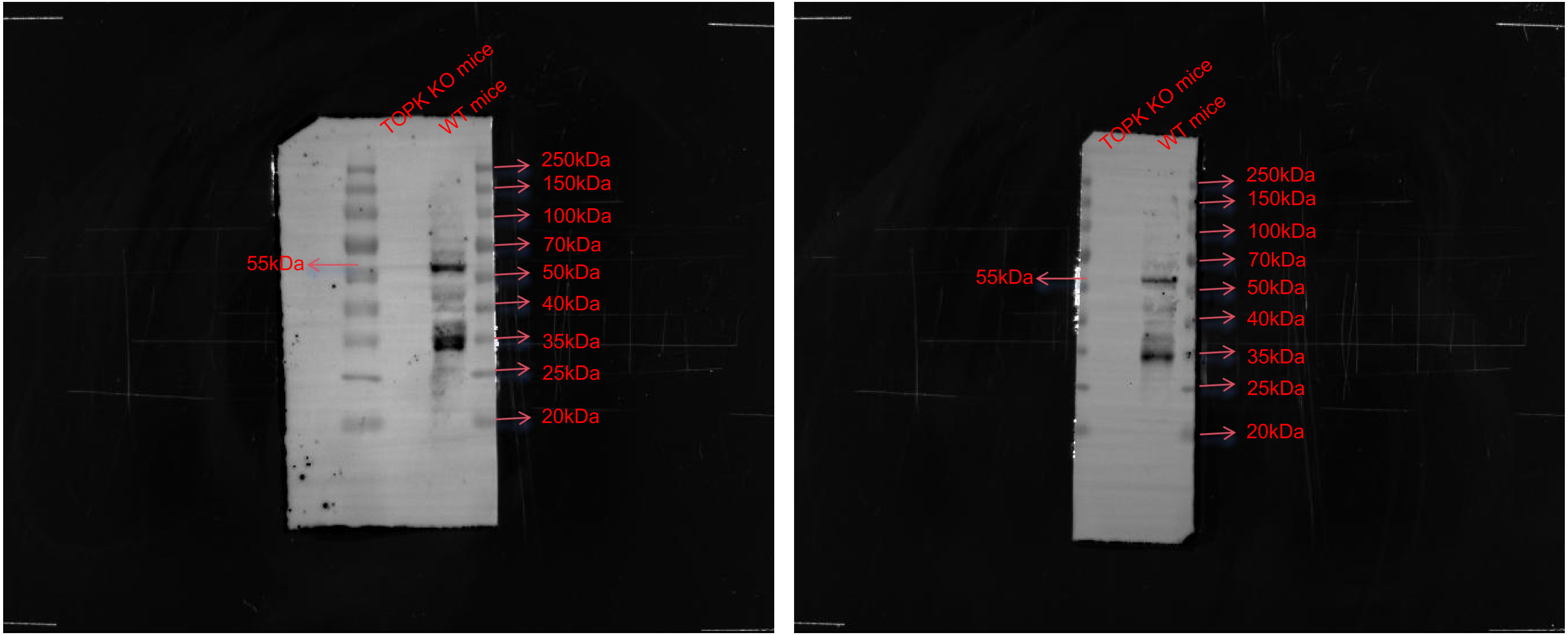

Fuller-length, original, and unprocessed Westem blot images of Anti- TOPK antibody to confim specific detection of the target antigen.(Predicted band size: 55 kDa Observed band size: 55 kDa)

Fig-7B TOPK

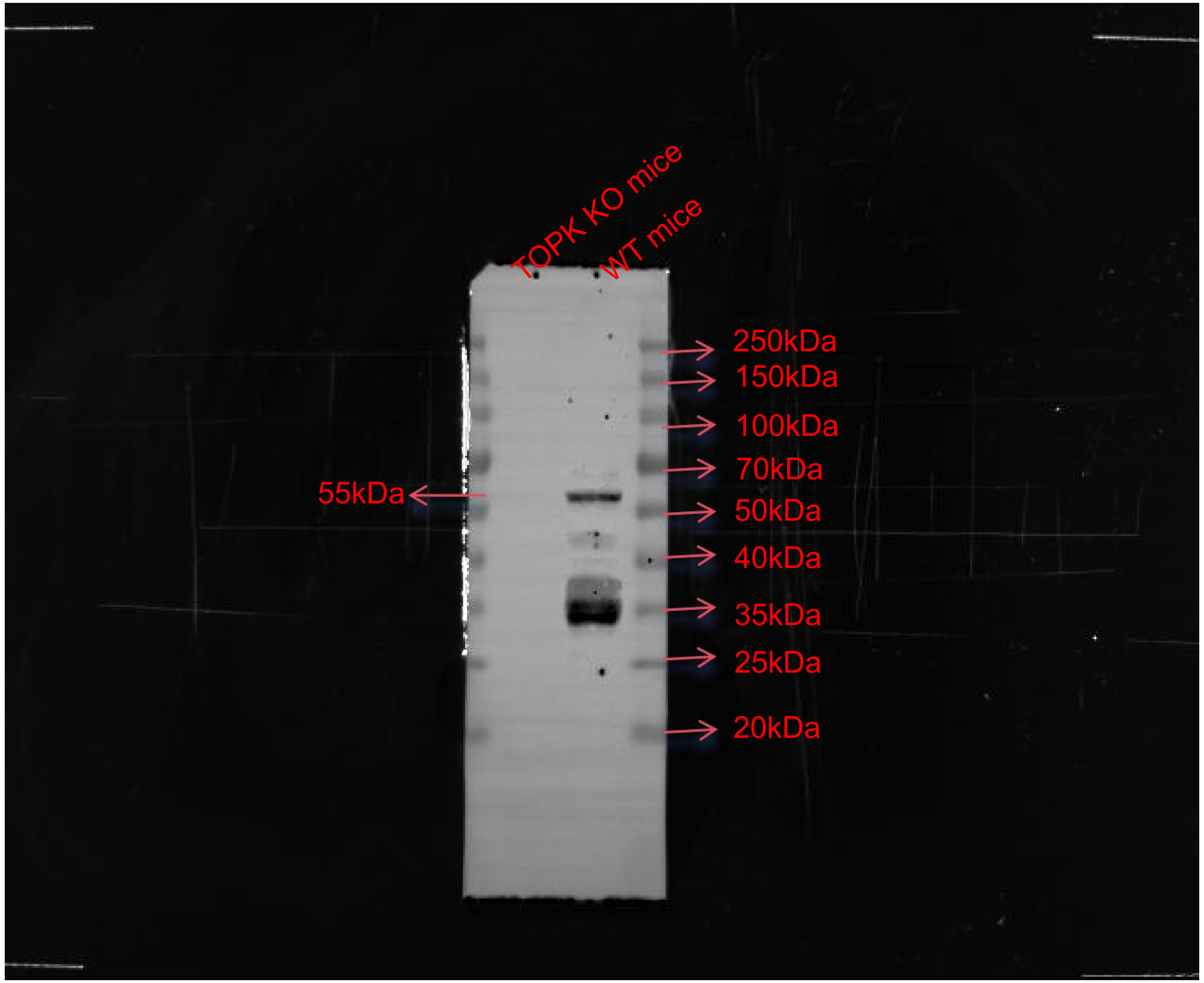

Fuller-length, original, and unprocessed Westem blot images of Anti- TOPK antibody to confim specific detection of the target antigen.(Predicted band size: 55 kDa Observed band size: 55 kDa)
